# A new polygenic score for refractive error improves detection of children at risk of high myopia but not the prediction of those at risk of myopic macular degeneration

**DOI:** 10.1016/j.ebiom.2023.104551

**Published:** 2023-04-11

**Authors:** Rosie Clark, Samantha Sze-Yee Lee, Ran Du, Yining Wang, Sander C.M. Kneepkens, Jason Charng, Yu Huang, Michael L. Hunter, Chen Jiang, J.Willem L. Tideman, Ronald B. Melles, Caroline C.W. Klaver, David A. Mackey, Cathy Williams, Hélène Choquet, Kyoko Ohno-Matsui, Jeremy A. Guggenheim, CREAM Consortium, Joan E. Bailey-Wilson, Paul N. Baird, Veluchamy A. Barathi, Ginevra Biino, Kathryn P. Burdon, Harry Campbell, Li Jia Chen, Ching-Yu Cheng, Emily Y. Chew, Jamie E. Craig, Margaret M. Deangelis, Cécile Delcourt, Xiaohu Ding, Qiao Fan, Maurizio Fossarello, Paul J. Foster, Puya Gharahkhani, Jeremy A. Guggenheim, Xiaobo Guo, Annechien E.G. Haarman, Toomas Haller, Christopher J. Hammond, Xikun Han, Caroline Hayward, Mingguang He, Alex W. Hewitt, Quan Hoang, Pirro G. Hysi, Adriana I. Iglesias, Robert P. Igo, Sudha K. Iyengar, Jost B. Jonas, Mika Kähönen, Jaakko Kaprio, Anthony P. Khawaja, Barbara E. Klein, Jonathan H. Lass, Kris Lee, Terho Lehtimäki, Deyana Lewis, Qing Li, Shi-Ming Li, Leo-Pekka Lyytikäinen, Stuart MacGregor, David A. Mackey, Nicholas G. Martin, Akira Meguro, Andres Metspalu, Candace Middlebrooks, Masahiro Miyake, Nobuhisa Mizuki, Anthony Musolf, Stefan Nickels, Konrad Oexle, Chi Pui Pang, Olavi Pärssinen, Andrew D. Paterson, Norbert Pfeiffer, Ozren Polasek, Jugnoo S. Rahi, Olli Raitakari, Igor Rudan, Srujana Sahebjada, Seang-Mei Saw, Claire L. Simpson, Dwight Stambolian, E-Shyong Tai, Milly S. Tedja, J. Willem L. Tideman, Akitaka Tsujikawa, Cornelia M. van Duijn, Virginie J.M. Verhoeven, Veronique Vitart, Ningli Wang, Ya Xing Wang, Juho Wedenoja, Wen Bin Wei, Cathy Williams, Katie M. Williams, James F. Wilson, Robert Wojciechowski, Jason C.S. Yam, Kenji Yamashiro, Maurice K.H. Yap, Seyhan Yazar, Shea Ping Yip, Terri L. Young, Xiangtian Zhou, UK Biobank Eye and Vision Consortium, Naomi Allen, Tariq Aslam, Denize Atan, Sarah Barman, Jenny Barrett, Paul Bishop, Graeme Black, Catey Bunce, Roxana Carare, Usha Chakravarthy, Michelle Chan, Sharon Chua, Valentina Cipriani, Alexander Day, Parul Desai, Bal Dhillon, Andrew Dick, Alexander Doney, Cathy Egan, Sarah Ennis, Paul Foster, Marcus Fruttiger, John Gallacher, David Garway-Heath, Jane Gibson, Dan Gore, Jeremy Guggenheim, Chris Hammond, Alison Hardcastle, Simon Harding, Ruth Hogg, Pirro Hysi, Pearse A. Keane, Peng Tee Khaw, Anthony Khawaja, Gerassimos Lascaratos, Thomas Littlejohns, Andrew Lotery, Phil Luthert, Tom MacGillivray, Sarah Mackie, Bernadette McGuinness, Gareth McKay, Martin McKibbin, Danny Mitry, Tony Moore, James Morgan, Zaynah Muthy, Eoin O'Sullivan, Chris Owen, Praveen Patel, Euan Paterson, Tunde Peto, Axel Petzold, Nikolas Pontikos, Jugnoo Rahi, Alicja Rudnicka, Jay Self, Panagiotis Sergouniotis, Sobha Sivaprasad, David Steel, Irene Stratton, Nicholas Strouthidis, Cathie Sudlow, Robyn Tapp, Caroline Thaung, Dhanes Thomas, Emanuele Trucco, Adnan Tufail, Stephen Vernon, Ananth Viswanathan, Veronique Vitart, Cathy Williams, Katie Williams, Jayne Woodside, Max Yates, Jennifer Yip, Yalin Zheng

**Affiliations:** a School of Optometry & Vision Sciences, Cardiff University, Maindy Road, Cardiff, CF24 4HQ, UK; b University of Western Australia, Centre for Ophthalmology and Visual Science (incorporating the Lions Eye Institute), Perth, Western Australia, Australia; c Department of Ophthalmology and Visual Science, Tokyo Medical and Dental University, 1-5-45 Yushima, Bunkyo-ku, Tokyo, 1138510, Japan; d Department of Ophthalmology, Beijing Children's Hospital, Capital Medical University, National Center for Children's Health, Beijing, 100045, China; e Department of Ophthalmology, Erasmus University Medical Center, Rotterdam, the Netherlands; f Department of Epidemiology, Erasmus University Medical Center, Rotterdam, the Netherlands; g Generation R Study Group, Erasmus University Medical Center, Rotterdam, the Netherlands; h Department of Optometry, School of Allied Health, University of Western Australia, Perth, Australia; i Department of Ophthalmology, Guangdong Eye Institute, Guangdong Provincial People's Hospital, Guangdong Academy of Medical Sciences, Guangzhou, 510080, China; j Busselton Health Study Centre, Busselton Population Medical Research Institute, Busselton, Western Australia; k School of Population and Global Health, University of Western Australia, Perth, Western Australia; l Division of Research, Kaiser Permanente Northern California, Oakland, CA, USA; m Department of Ophthalmology, Martini Hospital, Groningen, the Netherlands; n Department of Ophthalmology Kaiser Permanente Northern California, Redwood City, CA, USA; o Institute of Molecular and Clinical Ophthalmology, Basel, Switzerland; p Department of Ophthalmology, Radboud University Medical Center, Nijmegen, the Netherlands; q Centre for Eye Research Australia, Royal Victorian Eye and Ear Hospital, University of Melbourne, East Melbourne, Victoria, Australia; r School of Medicine, Menzies Research Institute Tasmania, University of Tasmania, Hobart, Tasmania, Australia; s Centre for Academic Child Health, Population Health Sciences, Bristol Medical School, University of Bristol, Bristol, BS81NU, UK

**Keywords:** Myopia, Polygenic score, UK Biobank, Generation R, ALSPAC

## Abstract

**Background:**

High myopia (HM), defined as a spherical equivalent refractive error (SER) ≤ −6.00 diopters (D), is a leading cause of sight impairment, through myopic macular degeneration (MMD). We aimed to derive an improved polygenic score (PGS) for predicting children at risk of HM and to test if a PGS is predictive of MMD after accounting for SER.

**Methods:**

The PGS was derived from genome-wide association studies in participants of UK Biobank, CREAM Consortium, and Genetic Epidemiology Research on Adult Health and Aging. MMD severity was quantified by a deep learning algorithm. Prediction of HM was quantified as the area under the receiver operating curve (AUROC). Prediction of severe MMD was assessed by logistic regression.

**Findings:**

In independent samples of European, African, South Asian and East Asian ancestry, the PGS explained 19% (95% confidence interval 17–21%), 2% (1–3%), 8% (7–10%) and 6% (3–9%) of the variation in SER, respectively. The AUROC for HM in these samples was 0.78 (0.75–0.81), 0.58 (0.53–0.64), 0.71 (0.69–0.74) and 0.67 (0.62–0.72), respectively. The PGS was not associated with the risk of MMD after accounting for SER: OR = 1.07 (0.92–1.24).

**Interpretation:**

Performance of the PGS approached the level required for clinical utility in Europeans but not in other ancestries. A PGS for refractive error was not predictive of MMD risk once SER was accounted for.

**Funding:**

Supported by the 10.13039/100015846Welsh Government and 10.13039/100002089Fight for Sight (24WG201).

**Research in context**

**Evidence before this study**

Quantifying a person's genetic susceptibility to refractive error by means of a polygenic score (PGS) has previously been proposed as a method to identify children at increased risk of high myopia who would benefit from early treatment intervention. Existing polygenic scores have lacked the accuracy required for clinical utility. It was already known that a PGS for refractive error is predictive of the risk of myopic macular degeneration (MMD). We carried out a literature search in Google Scholar for the keywords “myopia” and either “polygenic” or “genetic risk” to identify studies on this topic published in English, with no date restriction.

**Added value of this study**

We tested if a PGS for refractive error derived using state-of-the-art methods reached the level of accuracy required for clinical utility in predicting children at risk of high myopia. We also tested if a PGS for refractive error had clinical value in predicting individuals at increased risk of MMD once their level of myopia was accounted for.

**Implications of all the available evidence**

A state-of-the-art PGS has a level of accuracy approaching that required for clinical utility in predicting children at risk of high myopia, but only for children of European ancestry. A risk prediction model for MMD based on traditional risk factors was not improved by the inclusion of a PGS for refractive error.

## Introduction

Refractive error is a continuous ocular trait that quantifies the eye's capacity to achieve sharp distance vision when accommodation of the crystalline lens is relaxed.^1^, ^2^, ^3^ The negative arm of the refractive error distribution, referred to as myopia, corresponds to the need for diverging lens power in the form of spectacles, contact lenses or refractive surgery to achieve high acuity distance vision. At the opposite end of the distribution, hyperopia indicates the need for continuous accommodation or converging lens-power spectacles or contact lenses to achieve clear distance vision. Hyperopia and myopia are risk factors for strabismus and amblyopia,^4^^,^^5^ while myopia also increases the risk of retinal detachment, glaucoma and myopic macular degeneration (MMD).^6^ In a recent meta-analysis,^7^ the pooled prevalence of MMD across the world population was 2.1%, 95% confidence interval (CI) 1.3–3.3%. The prevalence of MMD is approximately twice as high in South and East Asia compared to other regions, a trend expected to continue in the future.^8^

Research comparing the concordance of myopia in pairs of monozygotic and dizygotic twins provided early evidence that refractive error is highly heritable.^9^^,^^10^ Recent molecular genetic studies have identified several disease genes harboring missense or loss-of-function mutations that cause monogenic high myopia or high hyperopia in isolated families.^11^, ^12^, ^13^ Meanwhile, population-based genome-wide association studies (GWAS) have identified hundreds of independent genetic risk variants, each of which confers a small increase in the risk of myopia and a decrease in the risk of hyperopia, or vice versa.^14^, ^15^, ^16^, ^17^, ^18^ Compared to the rare monogenic forms of high myopia or hyperopia, the polygenic contribution to refractive error is far less deterministic, with lifestyle risk factors playing a greater role.^19^, ^20^, ^21^, ^22^ Recent work suggests high myopia is associated with carrying an excess of polygenic “risk” variants or a rare monogenic mutation.^18^^,^^23^

The increasing burden to society from MMD, especially in East and South East Asia, coupled with the recent availability of pharmacotherapeutic and optical interventions to slow the progression of myopia during childhood,^24^, ^25^, ^26^, ^27^ has raised interest in the early detection of children at risk of developing high myopia.^28^, ^29^, ^30^ Profiling genetic susceptibility via a “polygenic score” (PGS) has been considered as a means of quantifying a child's future risk of myopia, high myopia, or MMD.^31^, ^32^, ^33^, ^34^, ^35^ The performance limit of such a PGS is governed by the “SNP-heritability” of refractive error, which represents the person-to-person variation in refractive error within a population explained by commonly-occurring genetic variants.^36^ The SNP-heritability of refractive error has been estimated at 35–45%.^37^^,^^38^ Currently, the best-performing PGSs for refractive error explain about 11% of the variance in the trait in individuals of European ancestry^33^ (about 25% of the SNP-heritability) and about 5% of the variance in those of East Asian ancestry.^35^^,^^39^ It is not known at present if a PGS for refractive error is predictive of MMD once a patient's refractive error is taken into account. In the current study, we derived an improved PGS for refractive error that explains 19% of the variance in refractive error in Europeans (about 50% of the heritability), 2% in African ancestry individuals, 6% in East Asian and 8% in South Asian ancestry individuals. We also applied a deep learning (DL) algorithm to grade MMD severity in 75,869 United Kingdom (UK) Biobank participants, which enabled us to test if the PGS was predictive of severe MMD after accounting for refractive error.

## Methods

### Ethics

The research adhered to the tenets of the Declaration of Helsinki. The UK Biobank study is a prospective cohort study of approximately 500,000 adults from across the UK investigating how genetics and lifestyle influence wellbeing and disease.^40^ Ethics approval was obtained from the UK National Health Service (NHS) Research Ethics Committee (Reference: 11/NW/0382). All participants provided written informed consent. The Genetic Epidemiology Research on Adult Health and Aging (GERA) study is a component of the Kaiser Permanente Research Program on Genes, Environment, and Health (RPGEH).^41^^,^^42^ GERA participants are an unselected cohort of 110,266 adult members of Kaiser Permanente Northern California (KPNC), an integrated healthcare delivery system. All participants provided written informed consent. The institutional review board of the Kaiser Foundation Research Institute approved the study procedures. The Consortium for Refractive Error and Myopia (CREAM) is an international collaborative organization of researchers investigating the genetics of refractive error. Ethics approval for participants enrolled in CREAM samples is described in Note S1 and the article by Tedja et al.^16^ All CREAM participants provided informed consent and all studies obtained ethics approval from their local Institutional Research Board or other local authorization body. The Busselton Healthy Ageing Study^43^ (BHAS) recruited a population-based sample of 5107 participants from the Busselton coastal community in Western Australia, who were born between 1946 and 1964. Ethics approval was obtained from the University of Western Australia Human Research Ethics Committee (Number 2021/ET000260). All participants provided informed consent. The Avon Longitudinal Study of Parents and Children (ALSPAC)^44^^,^^45^ recruited pregnant women resident in Avon, UK with expected dates of delivery 1st April 1991–31st December 1992. Attempts were made subsequently to bolster the initial sample with eligible cases who had failed to join the study originally, which resulted in an additional 913 children being enrolled. At the age of 1 year of age, 14,901 children were participating, along with their mothers or guardians. The study website contains details of all the data that is available through a fully searchable data dictionary and variable search tool: http://www.bristol.ac.uk/alspac/researchers/our-data/. Ethics approval for the study was obtained from the ALSPAC Ethics and Law Committee and the Local Research Ethics Committees (Refs: E1808/E4168/E5215/E5691/E5806/06/Q2006/53). Informed consent for the use of data collected via questionnaires and clinics was obtained from participants following the recommendations of the ALSPAC Ethics and Law Committee at the time. The Generation R study^46^^,^^47^ is a population-based prospective cohort study from fetal life to adulthood. A total of 9778 pregnant women resident in Rotterdam, The Netherlands, with a delivery date from April 2002 until January 2006 were recruited. The Generation R Study received ethics approval from the Medical Ethical Committee of Erasmus Medical Center, University Medical Center Rotterdam (Ref: MEC 217.595/2002/20). All participants provided written informed consent for each phase of the study (fetal, preschool, childhood and adolescence period). Children provided consent from the age of 12 years onwards, in accordance with Dutch Law.

### Definitions of hyperopia, myopia and high myopia

Following the convention of previous genetic studies,^15^, ^16^, ^17^ the refractive error of participants was based on autorefractor-measured spherical equivalent refractive error (SER) averaged between the two eyes (*avSER*). Myopia was defined as *avSER* ≤ −0.50 D, moderate myopia (MM) as *avSER* ≤ −3.00 D and moderate hyperopia (MH) as *avSER* ≥ +3.00 D. To allow comparison with earlier studies, two definitions for high myopia (HM) were used: *avSER* ≤ −6.00 D (HM6) and *avSER* ≤ −5.00 D (HM5). Refractive error measurement in each sample is described in Table 1 and Table S3. For young persons and adults in the ALSPAC, refractive error was measured by autorefraction *without* cycloplegia.

**Table 1:** Demographic characteristics of the European ancestry GWAS cohorts used to derive the PGS.

GWAS cohort	N	Age (years)	Female (%)	*avSER* (D)	Measurement method
UK Biobank GWAS for *avSER*	101,523	58.16 (7.96)	53.4	−0.28 (2.72)	Non-cycloplegic autorefraction
UK Biobank GWAS for *AOSW*-inferred *avSER*	290,188	57.93 (7.57)	55.0	Not available	Not applicable
CREAM GWAS for *avSER*	42,060	57.82 (14.15)	58.4	+0.00 (2.24)	Cycloplegic or non-cycloplegic autorefraction
GERA GWAS for *avSER*	34,998	66.54 (11.55)	58.7	−0.35 (2.56)	EMR (subjective refraction)

Values in brackets are standard deviations.

Abbreviations: *avSER* = Spherical equivalent refractive error averaged between the 2 eyes; *AOSW* = Age-of-onset of spectacle wear; EMR = Electronic medical records.

### Overview of the creation of the PGS for refractive error

As described in detail below, the multi-trait analysis of GWAS (MTAG) software^48^ was used to meta-analyze GWAS summary statistics for four non-overlapping samples of European ancestry: (1) a GWAS of *avSER* in 101,523 adult UK Biobank participants; (2) a GWAS of age-of-onset-of-spectacle-wear (*AOSW*)-inferred refractive error in 290,188 adult UK Biobank participants; (3) a GWAS meta-analysis of *avSER* in 42,060 adult Consortium for Refractive Error and Myopia (CREAM) consortium participants; (4) a GWAS of *avSER* in 34,998 GERA adults who self-reported as non-Hispanic white.^17^ Demographic characteristics of the four samples are given in Table 1. The MTAG meta-analysis results for a set of 1,035,607 SNPs were analyzed by LDpred2,^49^ with the settings optimized in an independent hold-out “tuning sample” of 4000 UK Biobank participants with known *avSER*. This resulted in a final PGS composed of approximately 770,000 SNPs. Full parameters of the PGS enabling its use by other researchers are available from https://doi.org/10.6084/m9.figshare.22294390.

### Selection of UK Biobank participants for GWAS and validation samples

Two definitions of European ancestry were applied: A “strict” definition and a “relaxed” definition. Participants were classified as meeting the relaxed definition of European ancestry if their first two genetic principal components (PCs) were within the mean ± 10 standard deviations of all unrelated UK Biobank participants who self-reported their ethnicity as “White British”.^50^ Participants were classified as meeting the strict definition of European ancestry if their first 20 PCs were within the mean ± 10 standard deviations for the “White British” group. Definitions for classifying UK Biobank participants as East Asian, South Asian or African ancestry were designed to maximize the available sample size while ensuring there was no overlap between the different ancestry groups (Figure S1). Participants were classified as East Asian if their first two PCs were within the mean ± 5 standard deviations of all UK Biobank participants who self-reported their ethnicity as “Chinese”. Participants were classified as South Asian if their first two PCs were within the mean ± 1.25 standard deviations of all UK Biobank participants who self-reported their ethnicity as “Asian”. Participants were classified as African if their first two PCs were within the mean ± two standard deviations of all UK Biobank participants who self-reported their ethnicity as “Black”. Applying these criteria, 978 participants of East Asian ancestry, 4641 participants of South Asian ancestry and 4089 participants of African ancestry were available and had autorefraction-measured refractive error information.

The GWAS selection scheme for European-ancestry UK Biobank participants is illustrated in Figure S2. First, a group of 56,917 participants was selected who met the “strict” criterion of European ancestry, were unrelated to any other individual in UK Biobank, had information for both autorefractor-measured *avSER* and self-reported *AOSW* and had no self-reported or hospital record report of an eye disorder that could affect refractive error (specifically, self-reported cataract, “serious eye problems”, “eye trauma”, cataract surgery, corneal graft surgery, laser eye surgery, or other eye surgery in the past 4 weeks, or a hospital record ICD10 code indicative of cataract surgery, eye surgery, retinal surgery, or retinal detachment surgery).^21^ From this group of 56,917 participants, a random selection of 10,000 was set aside as the “validation” sample. The “validation” sample was divided into a “tuning” sample (n = 4000) and a “test” sample (n = 6000). Second, a group of 101,523 participants were selected who were not in the “validation” sample, met the “relaxed” definition of European ancestry and had information for *avSER*. This sample was used for a GWAS for *avSER*. Third, a group of 50,000 participants were selected at random from those who were not in the “validation” sample, met the “strict” criterion of European ancestry, had information for both *avSER* and *AOSW* and had no self-reported or hospital record report of an eye disorder that could affect refractive error. This sample was used to derive a model to infer refractive error from *AOSW*:

Eq. 1 $$avSER\;∼\;poly\left(AOSW,10\right)+poly\left(Age,3\right)+Sex+\;poly\left(EduYears,2\right)+EduYears\times AOSW+\;PGS_{loco}+PGS_{loco}\times poly\left(AOSW,2\right)$$

where, poly (x,y) is an R function to compute orthogonal polynomials up to order *y* for variable *x*, *EduYears* is the participant's self-reported age of leaving full-time education (set as 21 years-old for those who reported having a College of University degree) and *PGS*_*loco*_ is a PGS created using the—predBetasFile function of BOLT^51^ for a sample of 51,523 participants of “relaxed” European ancestry not included in the validation sample or the 50,000 participants used to derive Eq. 1. In practice, *PGS*_*loco*_ was a set of 22 different PGSs, for each of which one of the 22 autosomes was omitted from the PGS (Figure S3). In turn, 22 different Eq.1 models were fit, each using a *PGS*_*loco*_ omitting one chromosome: a so-called leave-one-chromosome-out (LOCO) scheme. Fourth, a group of 290,188 participants was selected who were not in the “validation” sample, not in the sample of 101,523 participants used for the GWAS for *avSER*, met the “relaxed” definition of European ancestry and had information for *AOSW* and *EduYears*. Twenty-two separate GWAS analyses were carried out for this sample of 290,188 participants. In each case, the GWAS phenotype was *AOSW*-inferred refractive error, with the phenotype inferred from the values of *AOSW*, *Age*, *Sex*, *EduYears* and *PGS*_*loco*_ from Eq. 1 but using a different *PGS*_*loco*_ that omitted SNPs on one chromosome. A final set of GWAS summary statistics for the phenotype *AOSW*-inferred refractive error was created by taking the GWAS results for chromosome k (where k = 1, 2, 3 … 22) for the version of Eq. 1 fit using the *PGS*_*loco*_ that omitted chromosome k (Figure S3). This process ensured that genetic information from each chromosome was not used to help infer the relationship between *avSER* and *AOSW* when deriving that chromosome's contribution to the PGS for AOSW-inferred refractive error.

### GWAS procedures for UK Biobank samples

GWAS analyses were performed using BOLT v2.3.5.^51^ Age, age-squared, sex, genotyping array, and the first 10 genetic PCs were included as covariates. Genotype data in PLINK format were analyzed for the 1.03 million SNPs formed by intersecting the HapMap 3 variants used by default in the LDpred2^49^ and PRS-CS^52^ software (downloaded from https://figshare.com/articles/dataset/European_LD_reference_with_blocks_/19213299) and variants present in European UK Biobank participants with a genotyping call rate >0.95.

### Meta-analysis of GWAS summary statistics and creation of the PGS

Summary statistics from the GWAS for *avSER* in 101,523 UK Biobank participants, the GWAS for *AOSW*-inferred refractive error in 290,188 adult UK Biobank participants, the GWAS meta-analysis for *avSER* of 42,060 CREAM participants and the GWAS for *avSER* in 34,998 GERA participants were meta-analyzed with MTAG.^48^ Of the 1.03 million SNPs meta-analyzed, 958,542 SNPs were present in all four samples while 77,065 SNPs were present in the UK Biobank GWAS but missing from the CREAM or GERA summary statistics. In order for these 77,065 SNPs to be included in the PGS, a second MTAG meta-analysis was performed for just the two sets of UK Biobank GWAS summary statistics. Then the MTAG summary statistics for the 77,065 “missing” SNPs, obtained from the second MTAG meta-analysis, were added to the MTAG summary statistics for the remaining 958,542 SNPs.

LDpred2^49^ was used to account for linkage disequilibrium (LD) between markers, using the “tuning” sample to optimize the settings over a grid of parameters: heritability = {0.1, 0.2, 0.3, 0.4}; *P*-value threshold = {10^−5^, 10^−4^, 10^−3^, 10^−2^, 10^−1^, 1}; sparsity = {TRUE, FALSE}. Optimal accuracy in the tuning sample was obtained with the parameters: heritability = 0.3, *P*-value threshold = 10^−1^; sparsity = TRUE. The final PGS included 767,867 SNPs. A range of other methods was assessed for accounting for LD between markers; comparable accuracy was obtained for the mega-PRS (bld.ldak-tagging; BayesR) function of LDAK^53^ and lassosum,^54^ but none of the methods examined out-performed LDpred2.

### Creation of PGS for testing the association with MMD

To avoid bias when testing for an association between the PGS and MMD, it was necessary to derive a new PGS, because there was an overlap between (1) participants included in the GWAS for *avSER* in the UK Biobank sample, and (2) participants used to assess the association of the PGS vs. MMD. Accordingly, the 101,523 participants in the GWAS for *avSER* sample were divided into five approximately equal-sized tranches; this was done at random, except to ensure that no individual in a tranche was related to any person in the other four tranches (i.e. all related individuals were grouped in the same tranche). Next, leaving aside the first tranche, a GWAS for *avSER* was carried out for tranches two to five (approximately 80,000 participants). The resulting GWAS summary statistics were MTAG meta-analyzed with the other three sets of GWAS summary statistics (from the GWAS for *AOSW*-inferred refractive error and the CREAM and GERA GWAS analyses), processed by LDpred2, and used to derive a PGS for the participants in the first tranche. This process was repeated in turn for each of the other four tranches, thus providing an independent PGS for each of the 101,523 participants in the GWAS for *avSER* sample.

### Samples for assessing accuracy and predictive performance of the PGS for refractive error

The accuracy of the PGS was assessed initially in four “test” samples of adults from UK Biobank who were not related to, and did not overlap with, the participants included in the four GWAS analyses used to derive the PGS or a set of 4000 UK Biobank participants used as a “tuning” sample: (1) 6000 participants of European ancestry; (2) 4641 participants of South Asian ancestry; (2) 978 participants of East Asian ancestry; (3) 4089 participants of African ancestry. Performance was also assessed in three replication samples of European ancestry that were independent of UK Biobank: (1) Young persons from the Generation R study who underwent cycloplegic autorefraction at a research clinic when they were aged approximately 9 years-old and/or 13 years-old and had genotype data available.^55^ There were 1277 and 1649 participants with data available at age 9 and 13 years, respectively. Demographic characteristics of the Generation R samples are presented in Table S3. (2) Young persons from the ALSPAC who had their refractive error assessed by non-cycloplegic autorefraction longitudinally over childhood at the ages of 7, 10, 11, 12 and 15 years and had genotype data available.^37^ The sample size for participants with refractive error and genotype data ranged from 4037 to 6119 at each age point (Table S3). In total, 7177 children had information for at least one age point and 5566 had information at three or more ages; (3) 1476 mothers of ALSPAC children, who had non-cycloplegic autorefraction and genotype information available (Table S3).

### Statistics

Statistical analyses were carried out with R version 4.1.3. and the packages *boot* version 1.3.28, *pROC* version 1.18.0, *nlme* version 3.1.157, *lightgbm* version 3.3.2 and *logistf* version 1.24.1. Justification for the choice of statistical test is given in the relevant subsection below.

### Assessment of the accuracy of the PGS for refractive error

The PGS was standardized (to have a mean of zero and a standard deviation of one) in each ancestry group.^56^ The incremental R^2^, which corresponds to the proportion of variance in *avSER* explained by the PGS over that of a baseline model that adjusts for age, sex and genetic PCs, was calculated as the adjusted-R^2^ for a regression of *avSER* on a full set of covariates that included the PGS, minus the adjusted-R^2^ for a regression of *avSER* on the same set of covariates without the PGS. The full set of covariates comprised the PGS, age, age-squared, sex, genotyping array, and the first 10 genetic ancestry PCs. To account for sampling variability, the incremental R^2^ and its 95% CI were calculated as the median and 2.5th—97.5th percentiles from 2000 bootstrap replicates (R package *boot*).

### Assessment of the predictive performance of the PGS for high myopia and other categories of refractive error

To evaluate the performance of the PGS to predict myopia, MM, MH, HM5 and HM6, the area under the receiver operating characteristics curve (AUROC) was calculated, along with its 95% confidence interval from 2000 bootstrap replicates (R packages *pROC*). The relative performance two ROC models was compared using the bootstrap *roc. test* function, with 2000 bootstrap replicates. The AUROC corresponds to the expectation that a positive value drawn from a uniform distribution is ranked higher than a negative value when predicting the presence vs. absence of a specific type of refractive error, such as high myopia.

### Assessment of the accuracy of the PGS in the replication samples

The incremental R^2^ and AUROC for predicting a specific category of refractive error were calculated for young persons from the Generation R cohort and for young persons and adults from the ALSPAC using the methods described above for UK Biobank “test” samples. To evaluate the association of the PGS with the refractive error trajectory of children in the ALSPAC, linear mixed models (R package *nlme*) were fit as described.^57^ Participants were included if they had at least three refractive error measurements from clinic visits held when they were aged 7, 10, 11, 12 and 15 years-old. There were 5566 participants who met this criterion. Models were fit with *avSER* at each age point as the outcome variable. Age and higher-order age terms (age^2^ and age^3^), the PGS and an age × PGS interaction were modelled as fixed effects, while age nested within subject was modelled as a random effect, using an autoregressive correlation structure. The best-fit model was used to calculate the refractive error trajectory at specific quantiles of the PGS (5th, 25th, 50th, 75th and 95th percentile). For the ALSPAC samples, the current PGS for refractive error (“PGS 2022”) was compared in equivalent analyses of the same participants with two previously reported PGSs for refractive error: first, a PGS reported in 2018 (“PGS 2018”)^31^ that was derived from the 149 most strongly-associated independent SNPs identified in a UK Biobank GWAS for *avSER*; second, a PGS reported in 2020 (“PGS 2020”)^33^ that was previously the most accurate PGS for refractive error and was derived from 1.1 million SNPs located across the genome.

### Fundus images and quality grading of UK Biobank fundus images

Digital fundus photographs of both eyes were obtained for approximately 23% of UK Biobank cohort with a Topcon 3D OCT 1000 Mk2 instrument. There were 168,381 fundus images available in total. Table S1 shows the number of images obtained per eye. Forty-five degree angle digital fundus photographs centered on the fovea were obtained from both eyes of participants in the BHAS using a Canon CR-1 retinal camera. Participants with cataracts, a history of cataract surgery, or who did not have genotype data available were excluded.

A total of 4110 UK Biobank fundus images were selected at random from right or left eyes of participants of any ethnicity and manually labelled as good, poor or borderline image quality. Images with fully white or fully black fields of view were labelled as poor quality, as were images in which the majority of the fundus was too dark, too bright or too blurred to identify clinical features. Images in which clinical features could be identified over at least 50% of the field of view were considered as good quality; typically, the remaining area of the field of view was too dark to identify clinical features. Images that were not readily labelled as good or bad quality were labelled as borderline quality. Of the 4110 manually labelled images, 2741 were classified as good quality, 613 as poor quality and 756 borderline quality.

Image files were read using the R package *EBImage* and 21 different image features corresponding to the level of contrast, the mean intensity level or the standard deviation of the intensity level in defined areas of the image were extracted. A light gradient boosting machine decision tree (R package *lightgbm*)^58^ was trained to classify image quality as good or poor using these 21 features. Of the 2741 good quality and 613 poor quality labelled images, the algorithm was trained using 70% of the labelled image dataset, while the remaining 30% of the dataset was used for validation. In the validation sample, the algorithm's accuracy for detecting good quality images was 0.95 (95% confidence interval 0.94–0.97), with a sensitivity of 0.93 and a specificity of 0.96. The gradient boosting machine was used to grade image quality of all the remaining UK Biobank fundus images (completed in 3 h using 50 cores of a computing cluster). Only images with an image quality score >0.5 were included in the MMD analysis; approximately 15% of UK Biobank fundus images were excluded for having an image quality score below this threshold.

### Deep learning-based grading of MMD in UK Biobank and the BHAS

Meta-analysis of pathologic myopia (META-PM) grading of the UK Biobank and BHAS fundus images was carried out using the DL algorithm developed by Du et al.^59^ If present, MMD was graded as C2, C3 or C4, corresponding to diffuse atrophy, patchy atrophy and macular atrophy, respectively (Note S2). Expert ophthalmologist examination of the fundus images graded C3 or C4 from the BHAS led to the exclusion of 11 participants with probable toxoplasmosis rather than MMD. Expert ophthalmologist examination of a sample of UK Biobank images suggested that the DL algorithm was unreliable at detecting grade C2 MMD (diffuse atrophy). This was probably because the DL algorithm was trained using images from an East Asian cohort whereas most UK Biobank participants had European ancestry.^59^ By contrast, all images graded as C3 or C4 were confirmed by expert ophthalmologist examination to be consistent with a diagnosis of patchy atrophy and macular atrophy. As (i) the grading of MMD grade C2 was unreliable and (ii) few participants had MMD grade C3 or C4, the severity of MMD was further categorized as either, “Severe” (grade C3 or C4) or “Normal” (grade C0 or C2) when testing for factors associated with MMD.

### Samples for assessing the performance of the PGS in predicting MMD

UK Biobank participants were included in an analysis of factors associated with the presence of Severe MMD if they had information available for their genotypes, *avSER* and MMD-grade. For eyes with more than one high quality fundus image available, the image with the worse META-PM grade was taken as the META-PM grade for that eye. The META-PM grade of each participant was assigned as the worse grade in either the right or left eye. The same criteria were used to select a replication sample of BHAS participants. This yielded a sample of 75,869 UK Biobank participants and a sample of 4548 BHAS participants.

### Assessment of the performance of the PGS in predicting MMD

Variables associated with MMD grade in the worse eye were examined using multivariable logistic regression (R function *glm*). The outcome variable was the presence of Severe MMD (grade C3 or C4) or absence of MMD (grade C0 or C2) in the worse affected eye, while the predictor variables included the PGS, age, age-squared, sex, genotyping array, and the first 10 PCs. To test if the PGS was predictive of MMD independently of refractive error, the model was also fit with the inclusion of refractive error as a covariate. In view of the low number of Severe MMD cases, the logistic regression analyses were repeated using Firth bias-reduced logistic regression (R package *logistf*); the results were almost identical with standard and Firth bias-reduced logistic regression. For these tests of the relationship between the PGS and MMD, it was necessary to derive a distinct version of the PGS, in order to avoid overlap between the sample used to create the PGS and the sample used to evaluate its performance. The creation of this distinct version of the PGS is described above in the section, “*Creation of PGS for testing the association with MMD*”.

### Role of the funding source

The funders had no role in data collection, analysis, interpretation, writing, and the decision to submit.

## Results

Table 1 lists the demographic characteristics of the four GWAS cohorts used to create the PGS, all of which were of European ancestry. Table 2 presents the demographic characteristics of the four independent “test” samples of UK Biobank participants. These four “test” samples comprised participants of European, African, East Asian and South Asian ancestry, respectively.

**Table 2:** Demographic characteristics of the four independent “test” samples from UK Biobank.

Replication “test” sample	N	Age (years)	Female (%)	*avSER* (D)	Measurement method
European	5974	57.92 (7.53)	47.1	−0.32 (2.73)	Non-cycloplegic autorefraction
African	4089	52.82 (8.02)	59.5	−0.42 (2.32)	Non-cycloplegic autorefraction
South Asian	4641	54.31 (8.45)	48.3	−0.52 (2.49)	Non-cycloplegic autorefraction
East Asian	978	54.11 (8.04)	68.1	−1.53 (3.12)	Non-cycloplegic autorefraction

Values in brackets are standard deviations.

### Accuracy of the PGS for refractive error in independent “tuning” and “test” samples from UK Biobank

**Fig. 1:**
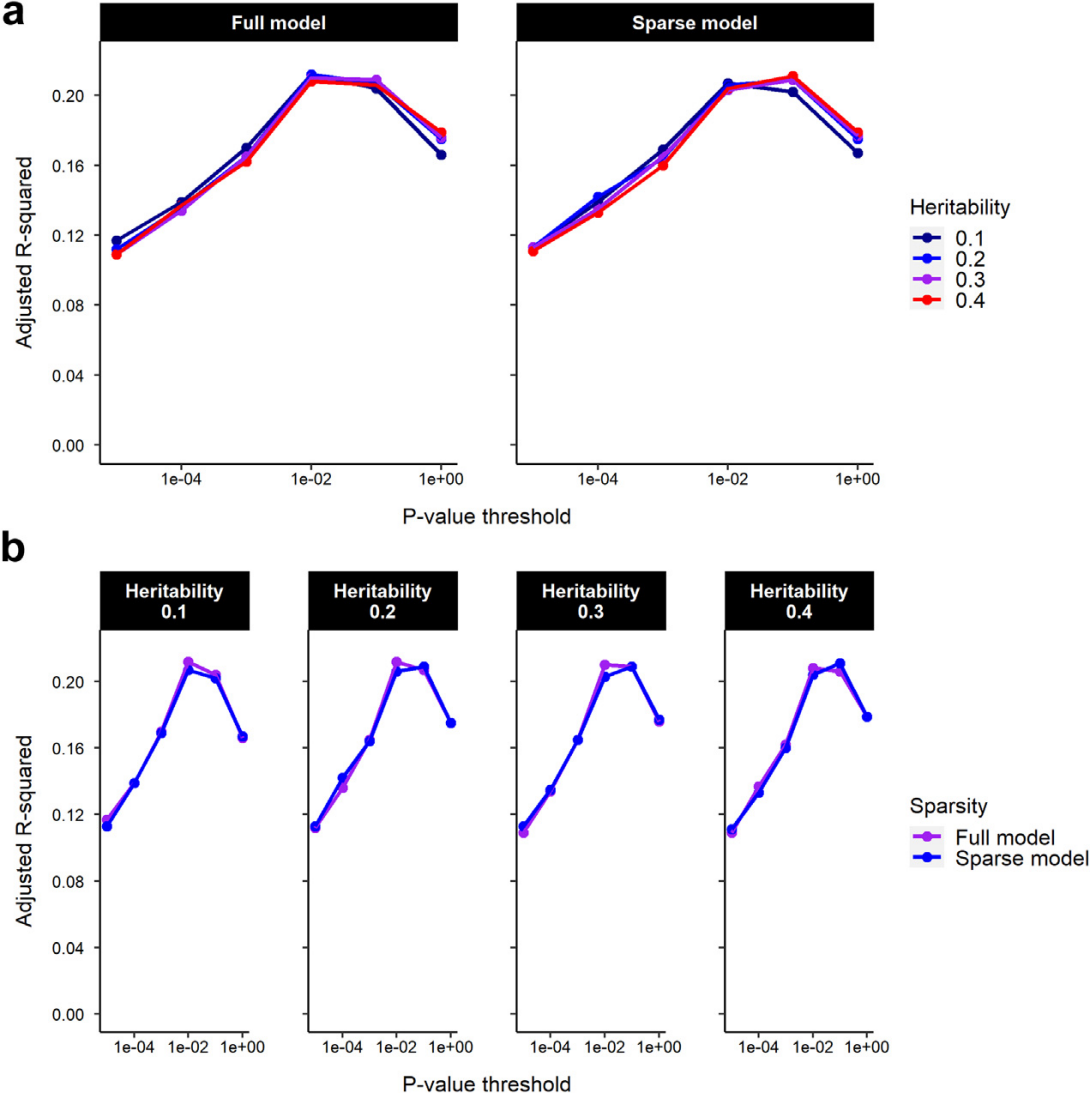
**Accuracy of the PGS over a grid of LDpred2 parameter settings in the “tuning” sample of4000 European-ancestry participants.** The model parameters varied were (**a**) model sparsity (full model vs. sparse model), (**b**) heritability (0.1, 0.2, 0.3 or 0.4). The p-value threshold from the GWAS meta-analysis summary statistics were varied across the range 1 × 10^−5^ to 1.0.

**Fig. 2:**
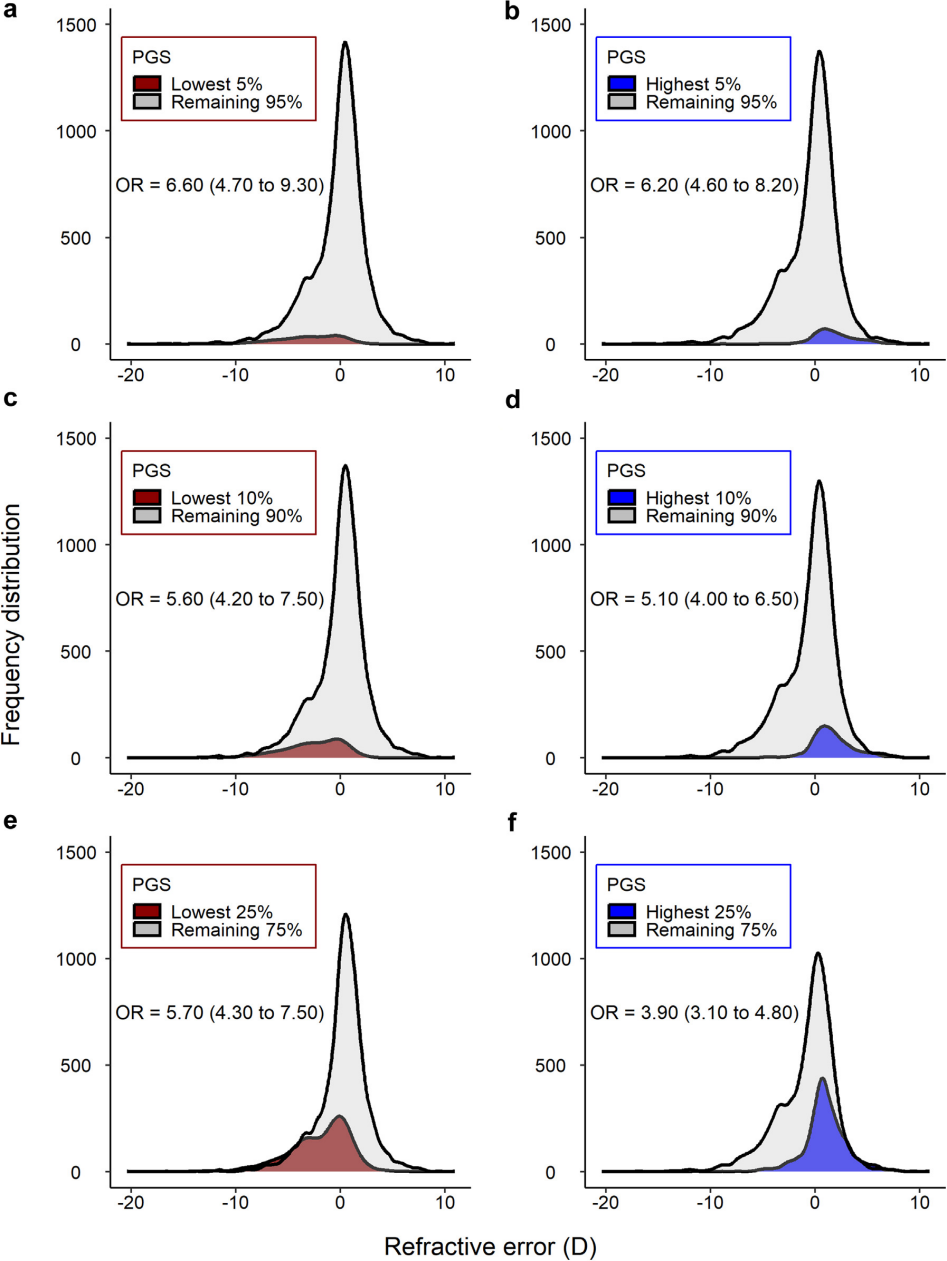
**Refractive error distribution in the “test” sample of 6000 European-ancestry particpants categorized as having a PGS above vs. below a threshold level.** (**a**, **c**, **e**) Odds ratio for predicting high myopia of −6.00D or worse. (**b**, **d**, **f**) Odds ratio for predicting moderate hyperopia of +3.00 D or worse (MH). The threshold levels examined were the top and bottom 5% (**a**, **b**), 10% (**c**, **d**) and 25% (**e**, **f**).

**Fig. 3:**
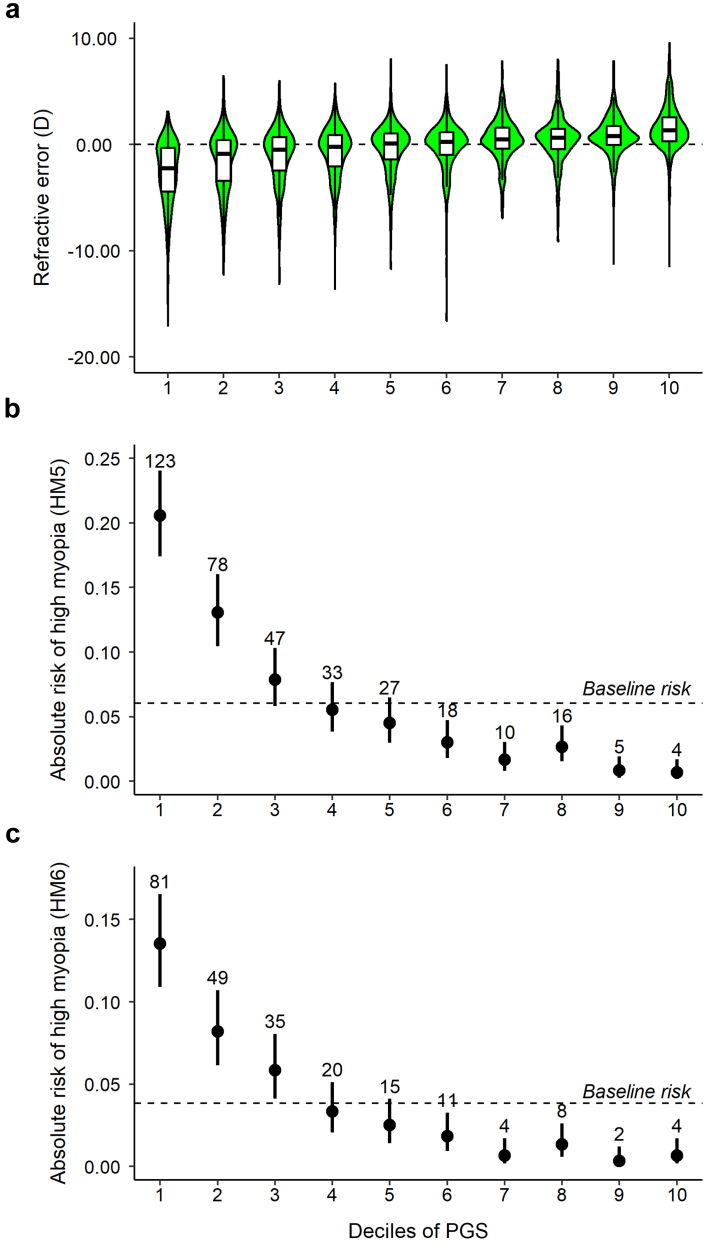
**Refractive error distribution and absolute risk of high myopia in deciles of the PGS, for the “test” sample of 6000 individuals of European ancestry. (a)** Distribution of refractive error by PGS decile. The white box corresponds to the interquartile range, the solid line inside the white box is the median. (**b**, **c)** Absolute risk of high myopia of at least −5.00 D (HM5; **b**) or of at least −6.00 D (HM6; **c**). Points correspond to the proportion of individuals in each decile affected by high myopia; error bars are 95% confidence intervals. Counts of affected individuals are shown above each point. The dashed horizontal line is the prevalence of high myopia in the full sample.

In a hold-out group of 4000 European-ancestry UK Biobank participants (“tuning” sample) who were unrelated to any person used in deriving the PGS, the PGS explained approximately 20% of the variance in refractive error under optimal tuning parameter settings (Fig. 1). The accuracy of the PGS in predicting refractive error in the four independent “test” samples is shown in Table 3 and Figs. 2 and 3. For the European ancestry “test” sample, the PGS had an incremental R^2^ = 0.19 (95% CI 0.17–0.21), representing a 70% improvement $\left(\frac{0.190-0.112}{0.112}\times 100\right)$ over the best-performing PGS for *avSER* reported previously.^33^ The PGS was much less accurate in UK Biobank “test” samples of non-European ancestry. The incremental R^2^ in the African, South Asian and East Asian “test” samples was 0.02 (95% CI 0.01–0.03), 0.08 (95% CI 0.07–0.10) and 0.06 (95% 0.03–0.09), respectively (Table 3). This represented a decrease in accuracy of 89%, 59% and 68% in the African, South Asian and East Asian samples compared to the European sample.

**Table 3:** Performance of the PGS in predicting refractive error, myopia and hyperopia in independent samples of UK Biobank participants.

Ancestry	Incremental R^2^	AUROC				
	Refractive error	Moderate hyperopia	Low myopia	Moderate myopia	High myopia (HM5)	High myopia (HM6)
European	0.190 (0.172–0.208)	0.742 (0.718–0.766)	0.728 (0.715–0.741)	0.754 (0.738–0.770)	0.774 (0.750–0.798)	0.783 (0.754–0.812)
African	0.020 (0.012–0.029)	0.610 (0.543–0.677)	0.582 (0.563–0.601)	0.596 (0.566–0.626)	0.586 (0.542–0.629)	0.584 (0.530–0.639)
South Asian	0.080 (0.066–0.096)	0.676 (0.631–0.721)	0.647 (0.631–0.664)	0.686 (0.664–0.708)	0.695 (0.664–0.726)	0.706 (0.668–0.743)
East Asian	0.060 (0.034–0.092)	0.610 (0.415–0.805)	0.653 (0.619–0.687)	0.660 (0.621–0.699)	0.648 (0.601–0.694)	0.672 (0.619–0.724)

Values in brackets are 95% confidence intervals. Samples sizes were: European (n = 6000); African (n = 4089); South Asian (n = 4641)’; East Asian (n = 978).

Abbreviations: Incremental R^2^ = Variance in refractive error explained by PGS; AUROC = Area under the receiver operating characteristics curve; HM5 = High myopia (≤−5.00 D); HM6 = High myopia (≤−6.00 D).

### Predictive performance of the PGS for high myopia in UK Biobank “test” samples

In the European “test” sample, the PGS had superior performance in detecting high myopia compared to the best previously reported PGS. For high myopia of −5.00 D or worse (HM5), the AUROC for the new PGS was 0.77 (95% CI 0.75–0.80) whilst for high myopia of −6.00 D or worse (HM6), the AUROC was 0.78 (95% CI 0.75–0.81). This compares to the best previous^33^ AUROC of 0.73 for HM5 (no AUROC for HM6 was reported previously^33^). The PGS also had the capacity to identify individuals with moderate hyperopia (AUROC = 0.74, 95% CI 0.72–0.77), and moderate myopia (AUROC = 0.75, 95% CI 0.74–0.77). When considering the full distribution of the PGS, ranging from negative (“low”) values indicating genetic susceptibility to myopia and positive (“high”) values indicating genetic susceptibility to hyperopia, individuals in the *lowest* 5%, 10% or 25% of the PGS distribution had a 6-fold–7-fold increased risk of HM6 compared to the remainder of the population (Fig. 2). By contrast, individuals in the *highest* 5%, 10% or 25% of the PGS distribution had a 4-fold–6-fold increased risk of moderate hyperopia (MH) compared to the remainder of the population (Fig. 2). In these individuals of European ancestry, the first two deciles of the PGS were highly enriched for participants with high myopia, while the last few deciles were highly depleted of participants with high myopia (Fig. 3). The AUROC for detecting individuals at risk of high myopia was worse in the non-European samples: the AUROC for both HM5 and HM6 in the African, South Asian and East Asian samples was <0.7 in all cases, except for HM6 in the South Asian sample (AUROC = 0.71, 95% CI 0.67–0.74; Table 3).

### Performance of the PGS in replication samples

**Fig. 4:**
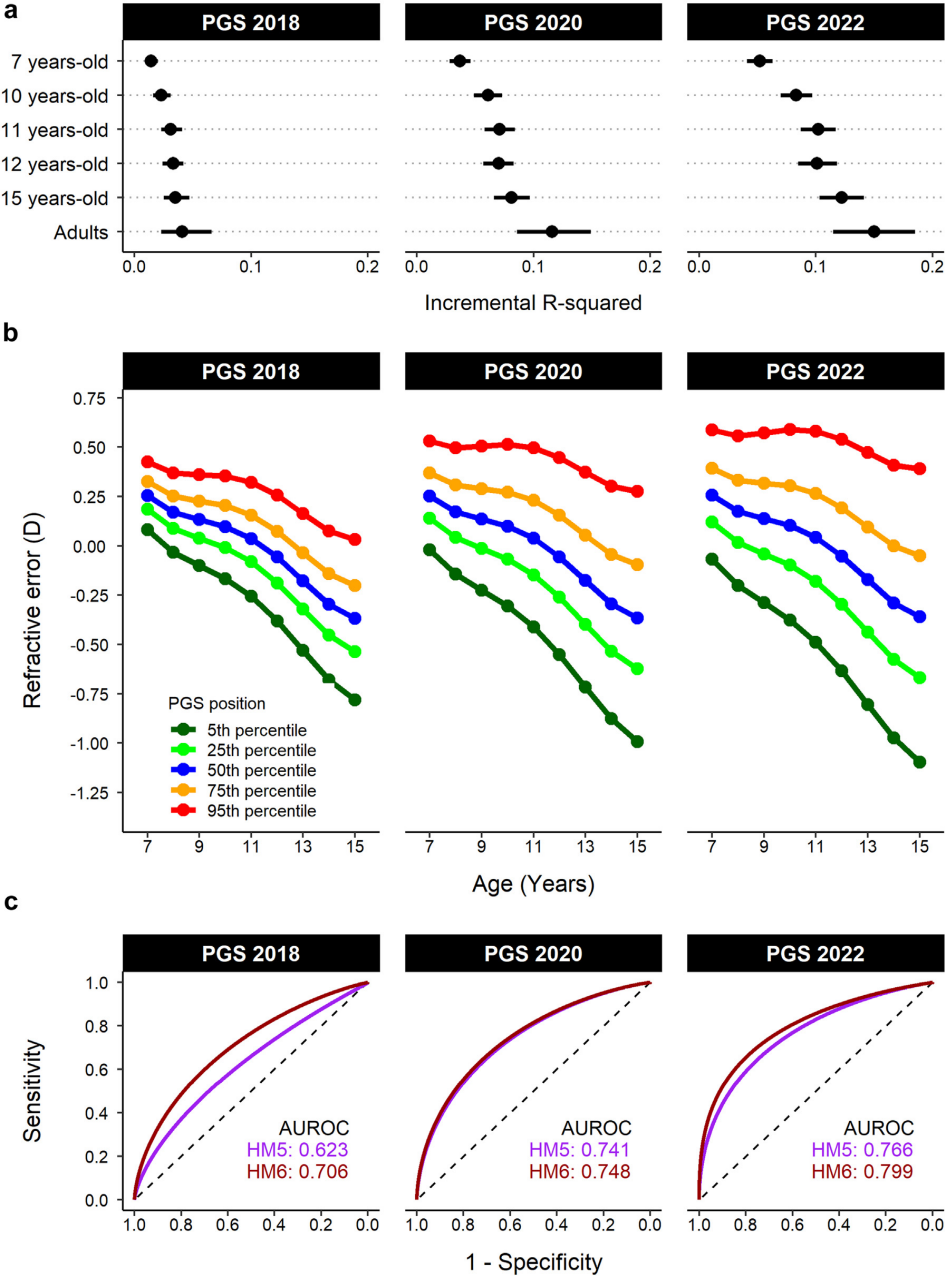
**Accuracy of the new and existing PGSs in independent samples of children and adults from the ALSPAC cohort.** Three different PGSs were compared: “PGS 2018” is a PGS created from the 149 SNPs most significantly associated with *avSER* in UK Biobank^31^; “PGS 2020” is a PGS created from 1.1 million SNPs associated with *avSER* or *AOSW*-inferred *avSER* in UK Biobank, which was previously the best-performing PGS for refractive error^33^; “PGS 2022” is the PGS reported in the current study. (**a**) Prediction accuracy of PGSs in young persons from the ALSPAC cohort whose refractive error was assessed longitudinally and in a sample of their mothers (adults). Error bars are 95% confidence intervals. (**b**) Refractive error trajectory of ALSPAC young persons, stratified by percentile of the PGS. (**c**) ROC curves for detecting high myopia (HM5 or HM6) in the adults from the ALSPAC cohort. The dashed black line represents chance-level prediction accuracy.

In young persons from Generation R, whose refractive error was assessed by cycloplegic autorefraction at age 9 years-old and 13 years-old, the PGS had an incremental R^2^ = 0.12 (95% CI 0.08–0.15) at age 9 years and 0.14 (95% CI 0.11–0.17) at age 13 years (Table 4). At age 9 years, prediction of HM5 was relatively poor (AUROC < 0.6) most likely due to the low prevalence of HM5 at this age (3 out of 1227 children; prevalence = 0.2%), which would make prediction more challenging.^60^ Prediction of HM6 at age 9 years was not possible due to an insufficient number of cases. By contrast, at age 13 years, performance in predicting high myopia was similar to that in adults of European ancestry (Table 4): AUROC for HM5 = 0.74 (95% 0.62–0.85) and AUROC for HM6 = 0.78 (95% 0.75–0.81). This suggests that the poor performance in detecting high myopia at age 9 years was due to children destined to become highly myopic not having a sufficient degree of myopia to be classified as affected at age 9. In young persons from the ALSPAC, whose refractive error was assessed longitudinally over childhood by non-cycloplegic autorefraction, the PGS had an incremental R^2^ = 0.05 (95% CI 0.04–0.06) at age 7 years that rose progressively with age to reach R^2^ = 0.12 (95% CI 0.10–0.14) at age 15 years (“PGS 2022” in Fig. 4a). When these participants were stratified by percentile of the PGS, then on average, there was a clear difference in refractive error trajectory across PGS percentiles (Fig. 4b). For adults from the ALSPAC sample, who were aged 44.22 ± 4.28 years (mean ± standard deviation) at the time their refractive error was measured, the PGS had an incremental R^2^ = 0.15 (95% CI 0.12–0.19). This sample of adults from the ALSPAC study was the same sample in which the previous best-performing PGS for refractive error (“PGS 2020”) had been reported.^33^ The PGS created in the current work (“PGS 2022”) attained a significant improvement in incremental R^2^ compared to PGS 2020 (Fig. 4a). Specifically, the increase in incremental R^2^ of PGS 2022 vs. PGS 2020 = 0.03 (95% CI 0.02–0.05) and far exceeded the performance of a PGS derived from the top genome-wide significantly associated GWAS SNPs (“PGS 2018”; Fig. 4a): increase in incremental R^2^ of PGS 2022 vs. PGS 2018 = 0.11 (95% CI 0.08–0.14). Prediction of HM5 was not significantly improved in the adult ALSPAC sample for PGS 2022 vs. PGS 2020 (bootstrap roc test: *P* = 0.15; PGS 2022 AUROC = 0.77, 95% CI 0.69–0.83; PGS 2020 AUROC = 0.74, 95% CI 0.67–0.81). However, prediction of HM6 was significantly improved with PGS 2022 compared to PGS 2020 (bootstrap roc test: *P* = 0.035; PGS 2022 AUROC = 0.80, 95% CI 0.69–0.88 and PGS 2020 AUROC 0.75, 95% CI 0.64–0.84).

**Table 4:** Performance of the PGS in predicting refractive, myopia and hyperopia in young persons from the Generation R cohort.

Clinic visit	Incremental R^2^	AUROC				
	Refractive error	Moderate hyperopia	Low myopia	Moderate myopia	High myopia (HM5)	High myopia (HM6)
9 years old	0.116 (0.079–0.153)	0.765 (0.695–0.825)	0.703 (0.654–0.751)	0.735 (0.600–0.854)	0.579 (0.567–0.592)	NA (only 1 case)
13 years old	0.141 (0.112–0.171)	0.774 (0.698–0.845)	0.691 (0.659–0.722)	0.691 (0.624–0.754)	0.739 (0.619–0.851)	0.783 (0.754–0.812)

Values in brackets are 95% confidence intervals. Samples sizes were: 9 year-olds visit (n = 1277); 13 year-olds visit (n = 1649).

Abbreviations: Incremental R^2^ = Variance in refractive error explained by PGS; AUROC = Area under the receiver operating characteristics curve; HM5 = High myopia (≤−5.00 D); HM6 = High myopia (≤−6.00 D).

### Performance of the PGS in predicting myopic maculopathy (MMD)

**Fig. 5:**
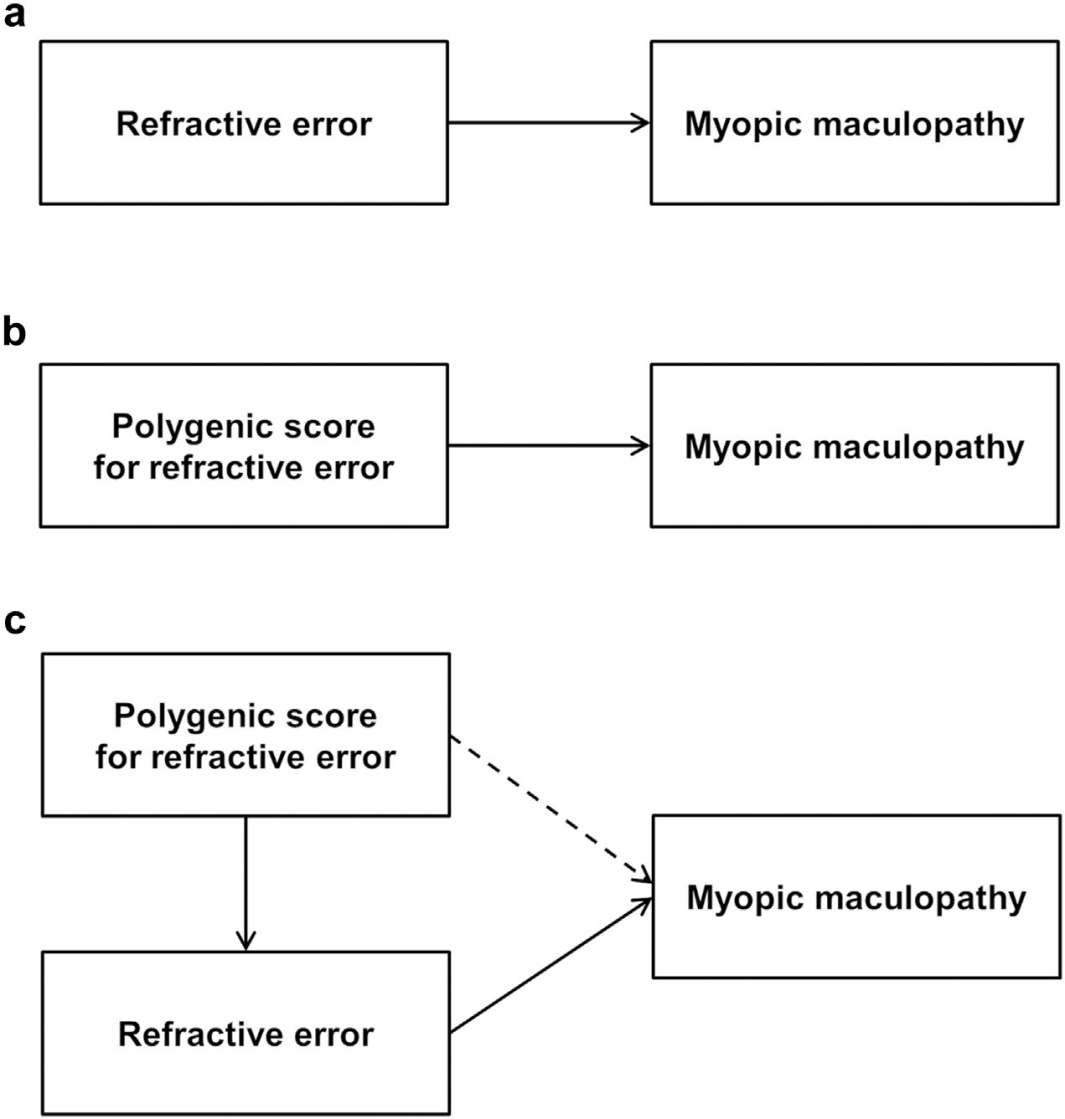
**Pathway diagrams (directed acyclic graphs) illustrating potential causal relationships between the PGS, refractive error, and myopic maculopathy.** (**a**) Refractive error is a major risk factor for MMD and it is highly plausible this relationship is causal. (**b**) A PGS for refractive error has a causal relationship with refractive error, therefore by logic, if refractive error is a cause of MMD then a PGS for refractive error must also be a cause of MMD. (**c**) While refractive error may be a mediator of the relationship between the PGS and MMD (solid arrows), it is possible that the PGS may confer an additional risk of MMD independently of the degree of refractive error (dashed arrow).

In total, 75,869 UK Biobank participants had gradable fundus images for at least one eye. The distribution of MMD in these participants, classified using the META-PM grading system, is presented in Table 5. Grade C3 MMD was detected in 169 (0.22%) and grade C4 MMD in 49 (0.06%) participants. We defined grade C3 or C4 MMD as “Severe”. Under the assumptions that (i) a PGS for refractive error has a causal relationship with refractive error and (ii) refractive error is a risk factor for MMD,^6^ it follows logically that a PGS for refractive error must be associated with MMD (Fig. 5a and b). However, it is possible that a PGS for refractive error confers an additional risk of MMD that is independent of the risk mediated by refractive error (Fig. 5c). To test this hypothesis, the relationship between the PGS and the presence of Severe MMD was assessed in a multiple regression analysis adjusting for age, sex, and genetic PCs. Confirming previous work,^6^ when the PGS was not included in the regression model, the degree of refractive error was strongly associated with the risk of Severe MMD: OR = 1.28 per diopter more negative refractive error (95% CI = 1.25–1.32; *P* = 3.61 × 10^−67^; logistic regression test). Also confirming previous work,^35^ when refractive error was not included in the regression model, the PGS was strongly associated with the risk of Severe MMD: OR = 1.56 per standard deviation of the PGS (95% CI 1.37–1.79, *P* = 1.04 × 10^−10^; logistic regression test). Crucially, when both refractive error and the PGS were included in the regression model, refractive error was still strongly associated with the risk of MMD grade C3–C4 (OR = 1.27 per diopter more negative refractive error, *P* = 3.59 × 10^−52^; logistic regression test) but the PGS was no longer associated (OR = 1.07 per standard deviation of the PGS, 95% CI 0.92–1.24, *P* = 0.37; logistic regression test). This finding suggests that all of the risk of MMD attributable to genetic susceptibility to refractive error is mediated by refractive error itself, i.e. with no risk along the path shown by the dashed arrow in Fig. 5c.

**Table 5:** Distribution of META-PM grade in the UK Biobank sample and the BHAS replication sample.

Grade	META-PM classification	Number of participants	
		UK Biobank	BHAS
C0	No myopic maculopathy	75,651	4533
C2	Diffuse atrophy	a	a
C3	Patchy atrophy	169 (0.22%)	10 (0.22%)
C4	Macular atrophy	49 (0.06%)	5 (0.11%)

Images were graded using a deep learning algorithm. The grade assigned to each participant was that in the worse-affected eye.

a Diffuse atrophy (grade C2) was reclassified as C0, due to unreliable grading of this category.

We attempted to replicate the MMD vs. PGS analysis in the independent BHAS sample. The demographic features of the BHAS sample are given in Table S3. Meta-PM grading of fundus photographs identified 10 BHAS participants with grade C3 and five participants with grade C4 MMD (Table 5). The prevalence of MMD grade C3 and C4 was similar in the UK Biobank sample and the BHAS sample (0.3% for grade C3 and C4 combined). The PGS explained approximately 7% of the variation in refractive error in the BHAS sample (incremental R^2^ = 0.069). The degree of refractive error was associated with the risk of Severe MMD in the BHAS sample: OR = 1.39 (95% CI 1.15**–**1.69, *P* = 0.0007 per diopter more negative refractive error; logistic regression test). However, the PGS was not associated with the risk of Severe MMD (OR = 1.20, 95% CI 0.72–1.99, *P* = 0.49, when refractive error was not included in the logistic regression model; OR = 1.00, 95% CI 0.60–1.66, *P* = 0.99 when refractive error was included in the logistic regression model; Table S4). The lack of an association between MMD grade and the PGS in the analysis not including refractive error was unexpected and implied that the analysis of the BHAS sample had insufficient statistical power to provide a valid test of replication.

## Discussion

This work presented an improved PGS for quantifying an individual's genetic susceptibility to refractive error. In independent samples from UK Biobank, the new PGS explained 19% of the variance in refractive error for Europeans, 2% in Africans, 6% in East Asians and 8% in South Asians. Maher^61^ suggested a predictive test should have an AUROC >0.8 to have clinical utility. Here, the PGS had higher predictive accuracy for high myopia than previously calculated PGSs, but fell just short of this level of accuracy, with an AUROC for HM6 of 0.78 in Europeans. Notably, the PGS was *not* predictive of MMD independently of refractive error, suggesting that all of the risk of MMD attributable to genetic susceptibility to refractive error is mediated by refractive error itself. Furthermore, predictive accuracy was worse for individuals of non-European ancestry (AUROC for HM6 of 0.58, 0.71 and 0.67 in Africans, South Asians and East Asians, respectively).

Strengths of the current study included the improved accuracy of the current PGS, which was derived from the meta-analysis of four separate large-scale GWAS studies across three different cohorts, the incorporation of information from approximately 770,000 SNPs, and an improved method for accounting for genetic variants in LD.^49^ Previous studies have derived PGSs using GWAS datasets with smaller sample sizes, or incorporated only the most significantly associated SNPs, or included myopia case–control GWAS studies as well as quantitative trait GWAS studies. An additional strength was the use of a DL algorithm to grade MMD severity in thousands of fundus images; accurate manual META-PM grading on this scale would have been challenging to achieve.

Despite analyzing fundus images for more than 75,000 individuals, the approximately population-based sampling of UK Biobank led to few participants with Severe MMD (grade C3 or C4) available for analysis. Nevertheless, there was extremely strong evidence for an association of the PGS with Severe MMD when the refractive error of participants was not considered (OR = 1.56, *P* = 1.04 × 10^−10^; logistic regression test). However, accounting for refractive error shifted this association decisively towards the null (OR = 1.07, *P* = 0.37; logistic regression test). This result provides robust evidence that even a powerful PGS for refractive error will not be clinically useful in predicting patients at risk of MMD; instead, SER remains a more easily measured predictor that is at least as accurate as the current PGS. Our attempt to replicate the MMD vs. PGS analysis in the BHAS was unsuccessful, as the analysis was underpowered to provide a valid replication for the UK Biobank analysis.

Screening for MMD in patients with high myopia would allow for early interventions to delay or prevent the development of irreversible visual impairment.^35^^,^^36^ Importantly, the current findings do not rule out the possibility that a PGS for MMD—rather than a PGS for SER—could have clinical utility in predicting a patient's risk of MMD. To derive a PGS for MMD it will be necessary to carry out a GWAS for MMD severity in a very large case-control sample; currently, no such sample is available. In the future, large-scale studies collecting fundus images for identification of MMD would benefit from careful quality control, to minimize the number of ungradable images. In the current study, 15% of UK Biobank fundus images could not be graded due to poor image quality. As an alternative to a PGS for refractive error, a PGS for axial length may have advantages for predicting MMD, since axial length provides a more direct index of posterior segment structural change. For instance, the crystalline lens refractive index increase accompanying age-related cataract development can shift SER by several diopters yet without altering axial length. However, axial length GWAS analyses in very large samples will be required to test this approach and such samples are not available at present.^62^

Studies of young persons in the Generation R and ALSPAC cohorts demonstrated that the accuracy of the PGS improved as children became older. For 13 year-old participants in the Generation R study, accuracy was already approaching the performance observed in adults, for example the AUROC for HM6 was 0.78 at this age. The use of non-cycloplegic autorefraction in the ALSPAC study may have led to the accuracy of the PGS being underestimated when tested in this cohort, since cycloplegic refraction is known to be more accurate than non-cycloplegic autorefraction at the ages these measurements were taken.

The improved PGS described here was less accurate in predicting high myopia than some existing methods based on measuring a child's SER. Chua et al.^63^ reported that in children aged 7–9 years, using age-of-onset of myopia as a predictor had an AUROC of 0.85 for predicting HM5 at age 11 years. Chen et al.^64^ reported that having an SER lower than the 5th percentile at age 7 years had 50% sensitivity and 100% specificity for predicting HM6 at age 15 years. However, these two studies did not assess accuracy in independent hold-out samples, therefore performance may have been overestimated due to over-fitting of the prediction model. Furthermore, these two studies only considered prediction of high myopia a few years into the future. By contrast, in a study that did evaluate performance in an independent validation sample, Lin et al.^65^ reported that for children examined at least 3 times between the ages of 8 and 15 years, a model based on the three predictors (i) age-at-examination, (ii) cycloplegic SER, and (iii) annual SER progression rate, had an AUROC of up to 0.88 for predicting HM6 over the next 5 years and an AUROC of 0.77 for predicting HM6 over the next 10 years. In another study that evaluated performance in an independent validation sample, Chen et al.^32^ obtained an AUROC of 0.80 for predicting the development of HM6 by the age of 18 years using a prediction model based on cycloplegic SER measured at age 9 years. These authors also achieved an AUROC > 0.95 for predicting the development of HM6 at age 18 years, with a prediction model based on cycloplegic SER measured at least twice prior to age 13 years. However, the key advantage of a PGS in this context is that it can be applied very early in life to identify children with an increased risk of high myopia much earlier than would be possible using cycloplegic autorefraction. Interest is growing in interventions designed to reduce the incidence of myopia, as opposed to slowing progression in existing myopia.^66^ For example, in Singapore, the ATOM-III study is currently evaluating the efficacy of atropine eye drops as a prophylactic intervention for myopia. Therefore, a PGS would have a unique advantage over competing methods in identifying children who would benefit from an intervention to reduce the risk of incident myopia.

It is well documented that prediction accuracy of a PGS derived from European training data is greatly reduced in samples of non-European ancestry,^35^^,^^67^ consistent with the results of the current study. Small improvements in the predictive power of the PGS in the non-European “test” samples from UK Biobank were observed compared to previously published PGSs, but there was still a decrease in accuracy of 89%, 59% and 68% in the African, South Asian and East Asian samples compared to the European sample. This reduced accuracy is known to be driven by differences in LD, allele frequency and causal variant effect-size across ancestries.^67^ This shortfall in performance in persons of non-European ancestry could exacerbate health disparities if a PGS was used in the clinic. A GWAS for SER in 100,000 or more participants of non-European ancestry is needed urgently to address this issue. Novel methods for deriving PGSs have sought to address the cross-ancestry transferability problem by focusing on putative causal variants.^68^, ^69^, ^70^, ^71^, ^72^ This approach has been partially successful in narrowing the cross-ancestry performance gap for PGSs that perform poorly in Europeans, but improvements for PGSs that perform well in Europeans are generally very limited. In the current study, the accuracy of the PGS in European samples varied widely, with an incremental R-squared of 19% (95% CI 17–21%) in UK Biobank, 15% (95% CI 12–19%) in ALSPAC adults, and 7% (95% CI 5.5–8.5%) in the BHAS. While this may partially represent sampling variation due to small sample sizes, it may also be caused by differences between these populations as regards the contribution to refractive error from genetic nurture (also known as “dynastic effects”^73^), assortative mating, gene-environment interactions^74^ and active gene-environment correlation (a type of “geographic effect”^75^). A recent study in UK Biobank participants suggested the contribution to the SNP-heritability of refractive error made by genetic nurture and assortative mating was minimal.^76^ Differences across populations in the degree of myopia gene-environment interaction have rarely been studied.^16^ Active gene-environment correlation can arise when individuals who share phenotypic features migrate from their place of birth to live together in neighborhoods.^75^ Thus, for example, the lower accuracy of the PGS in the ALSPAC adult sample compared to the UK Biobank European “test” sample could potentially be due to the ALSPAC adults being recruited from a single region of the UK, in which case the PGS would not be able to explain any variance in SER that arose due to region-to-region clustering of the SER phenotype across the UK (for which some evidence exists^20^).

In summary, a PGS quantifying genetic susceptibility to refractive error showed improved accuracy in predicting high myopia compared to the best previously published PGS.^33^ Although the current PGS was a significant improvement, accuracy fell just short of the level required for use in clinical practice to identify children at future risk of high myopia. Thus, cycloplegic autorefraction remains the recommended approach for screening for children at risk of future high myopia. Nonetheless, an advantage of a PGS is that children can be screened at an early age, and additional time outdoors even from the age of 3 years old is associated with a reduced incidence of myopia.^77^ The PGS was not predictive of MMD independently of refractive error, which implies that existing screening and monitoring methods remain the recommended approach for the early detection of MMD. Large GWAS studies of MMD are needed to further understand the complexity of this phenotype and to predict individuals who are most at risk.

## Contributors

CW, JAG and KOM conceived and designed the project. CJ, JAG, JC, RC, RD, SCMK, SSYL and YW performed the data analysis. CCWK, CW, DAM, HC, JAG, JWLT, KOM, MLH and RBM provided supervision and arranged access to datasets. YH provided methodological advice. CJ, CCWK, CW, DAM, HC, JAG, JWLT, RC, RBM, SCMK, SSYL and YH contributed to interpretation of data analysis results. RC, JAG, MLH, RD, SCMK and SSYL had access to the raw data sets and verified the data. RC and JAG wrote and prepared the Article. All authors had access to the data presented, read, revised and approved the Article.

## Data sharing statement

UK Biobank data are available via application at https://www.ukbiobank.ac.uk. Data for the Avon Longitudinal Study of Parents and Children (ALSPAC) cohort are available via application at http://www.bristol.ac.uk/alspac/. Data for the Generation R study are available via application for collaboration at https://generationr.nl/researchers/.

## Declaration of interests

The authors declare no potential conflicts of interests relevant to this manuscript. Outside this manuscript, KOM reports consultancy service for Santen and CooperVision; JAG reports membership of the Data Safety Monitoring Board for ‘CHAMPS-UK’ trial of atropine eyedrops for myopia (unpaid) and editorial board service for IOVS, TVST and OPO (unpaid).

## Appendix A Supplementary data

Tables S1–S4 and Figures S1–S3

CREAM Consortium list of names

UK Biobank Eye and Vision Consortium list of names
