# Supplementary material for: A new polygenic score for refractive error improves detection of children at risk of high myopia but not the prediction of those at risk of myopic macular degeneration
Source: eBioMedicine. 2023 Apr 11;91:104551. doi: 10.1016/j.ebiom.2023.104551 (PMC10203044; doi:10.1016/j.ebiom.2023.104551)
Supplement: Tables S1–S4 and Figures S1–S3 [file mmc1.pdf]

A new polygenic score for refractive error improves detection of children at risk of high myopia but not the prediction of those at risk of myopic macular degeneration.

Supplementary Information

Contents

Note S1. CREAM Consortium cohorts 1
Note S2. MMD grading criteria 1
Table S1. Number of fundus images per eye for UK Biobank participants. 2
Table S2. CREAM cohorts included in the current CREAM GWAS meta-analysis 3
Table S3. Demographic characteristics of replication samples. 4
Table S4. Test for association of MMD grade and PGS for refractive error in the BHAS sample. 5
Figure S1. Classification of ancestry groups. 6
Figure S2. Selection Scheme for UK Biobank participants. 7
Figure S3. Flow diagram describing the leave-one-chromosome-out (LOCO) scheme........ 8
References 9
CREAM Consortium members 10
UK Biobank Eye and Vision Consortium members........ 13

Note S1. CREAM Consortium cohorts

Ethical approval for the CREAM Consortium studies and details of recruitment, phenotyping, genotyping and imputation are described in Tedja et al. [1]. For the current study, the originally-reported refractive error GWAS meta-analysis was repeated. In contrast to the original CREAM meta-analysis described by Tedja et al., the new meta-analysis did not include participants in the ALSPAC Study and did not use genomic control correction. The meta-analysis was conducted using GWAMA [2]. CREAM cohorts included in the new meta-analysis are listed in Table S2 (available at www.aaojournal.org).

Note S2. MMD grading criteria

The following grading criteria were adopted:

C2 (Diffuse atrophy) was graded as diffuse chorioretinal atrophy. The tessellated change within the lesion disappears, and any choroidal vessels smaller than medium size also become unclear. The size of the atrophic area is over one disc area. It is not typical that definite diffuse chorioretinal atrophy occurs only around the optic disc or the total area is less than one disc area. Under this situation, the image was not graded as C2.

C3 (Patchy atrophy) was graded as patchy atrophy (peripapillary atrophy or patchy merged with peripapillary atrophy were not included as patchy atrophy). Patchy chorioretinal atrophy appears as a grayish-white, well defined lesion. Its size varies from one to several choroidal lobules in diameter and it appears in the macular area or around the optic disc. This small lesion looks brighter than its surroundings, or it may appear grayish white.

C4 (Macular atrophy) was graded as macular atrophy (atrophic lesion showed in macular or even fovea). A well-defined round chorioretinal atrophic lesions, which is dark green or grayish white.

Table S1. Number of fundus images per eye for UK Biobank participants.

Some UK Biobank participants had more than one image available, due to repeat fundus photography if the initial image was poor quality and/or if the participant underwent fundus photography at both the baseline visit and a follow-up imaging visit.

Eye	Number of images per eye		
	1	2	3
Right	78445	2894	21
Left	78246	2888	21

Table S2. CREAM cohorts included in the current CREAM GWAS meta-analysis

Cohort	Cohort name in full	Country	Sample size	Age (years)	Female (%)	*avSER* (D)
1958 BBC	1958 British Birth Cohort	United Kingdom	1 658	42.00 (0.00)	46.0	-0.96 (2.00)
ALIENOR	ALIENOR	France	509	79.15 (4.06)	56.8	+0.98 (1.97)
ANZRAG	Australia & New Zealand Registry Adv. Glaucoma	Australia	648	79.02 (12.08)	49.3	-0.21 (2.41)
AREDS	Age-Related Eye Disease Study	United States	1 842	68.08 (4.71)	59.0	+0.54 (2.16)
BATS	Brisbane Adolescent Twins Study	Australia	158	26.52 (2.41)	56.3	-0.51 (1.15)
BMES	Blue Mountains Eye Study	Australia	1 896	67.09 (9.16)	57.3	+0.62 (2.12)
CROATIA-KORCULA	Croatia Korcula Study	Croatia	822	56.33 (13.34)	64.8	-0.15 (1.60)
CROATIA-SPLIT	Croatia Split Study	Croatia	344	51.95 (13.02)	61.1	-1.27 (1.57)
CROATIA-VIS	Croatia Vis Study	Croatia	527	56.29 (13.30)	60.0	-0.13 (1.74)
DCCT	Diabetes Control and Complications Trial	United States	791	31.43 (4.13)	43.2	-1.47 (0.80)
EGCUT	Estonian Genome Center, Univ. of Tartu	Estonia	904	56.00 (17.00)	61.2	+0.33 (3.36)
EPIC-Norfolk	European Prospective Invest. into Cancer	United Kingdom	1 084	68.81 (7.55)	56.3	+0.34 (2.27)
ERF	Erasmus Rucphen Family Study	The Netherlands	2 610	48.72 (14.17)	55.0	+0.13 (2.03)
FECD	Fuchs' Endothelial Corneal Dystrophy Controls	United States	393	71.50 (9.18)	60.2	-0.14 (2.49)
FITSA	Finnish Twin Study on Aging	Finland	329	68.56 (3.35)	100.0	+1.22 (1.71)
FRAM	Framingham Eye Study	United States	2 729	55.60 (8.90)	42.5	+0.03 (2.41)
GHS1	Gutenberg Health Study-1	Germany	2 738	55.52 (10.81)	48.6	-0.38 (2.45)
GHS2	Gutenberg Health Study-2	Germany	1 140	54.81 (10.81)	50.4	-0.41 (2.57)
KORA	Cooperative Health Research in Region Augsburg	Germany	2 372	55.14 (11.79)	67.0	-0.25 (2.22)
OGP	Ogliastra Genetic Park Talana Study	Italy	509	51.43 (19.51)	59.2	-0.10 (1.67)
ORCADES	Orkney Complex Disease Study	United Kingdom	1 165	55.83 (13.76)	61.0	+0.09 (2.07)
RSI	Rotterdam Study-1	The Netherlands	5 787	68.84 (8.84)	59.4	+0.83 (2.55)
RSII	Rotterdam Study-2	The Netherlands	2 038	64.24 (7.75)	54.4	+0.49 (2.49)
RSIII	Rotterdam Study-3	The Netherlands	2 950	56.91 (6.54)	55.9	-0.28 (2.60)
TWINSUK	Twins UK Study	United Kingdom	4 342	53.83 (11.12)	92.2	-0.34 (2.72)
WESDR	Wisconsin Epidem. Study of Diabetic Retinopathy	United States	295	34.63 (8.05)	51.2	-1.53 (2.02)
YFS	Young Finns Study	Finland	1 480	41.94 (5.02)	55.4	-1.02 (1.99)
**Total**			**42 060**	**57.82 (14.15)**	**58.4**	**+0.00 (2.24)**

Table S3. Demographic characteristics of replication samples.

**Sample**	N	Age (years) mean ± SD (range)	Female N (%)	*avSER* mean ± SD	*Assessment of refractive error*
BHAS	4548	57.63 ± 5.71 (45.40–69.80)	2481 (54.6%)	+0.27 ± 1.54	Cycloplegic autorefraction (1% tropicamide) (ARK-30 instrument; Nidek, Japan)
Generation R children at ’9-year visit’	1277	9.83 ± 0.35 (8.87–11.98)	633 (49.6%)	+0.82 ± 1.23	Cycloplegic autorefraction (1% cyclopentolate) (Topcon KR8900 instrument; Topcon, Japan)
Generation R children at ’13-year visit’	1649	13.62 ± 0.36 (12.59–17.06)	846 (51.3%)	+0.39 ± 1.60	Cycloplegic autorefraction (1% cyclopentolate) (Topcon KR8900 instrument; Topcon, Japan)
ALSPAC children at ‘7-year visit’	6119	7.53 ± 0.31 (6.83–9.42)	3014 (49.3%)	+0.20 ± 0.88	Non-cycloplegic autorefraction (Canon R50 instrument, Canon Inc., USA)
ALSPAC children at ‘10-year visit’	5822	10.64 ± 0.25 (9.83–12.25)	2942 (50.5%)	+0.07 ± 1.10	Non-cycloplegic autorefraction (Canon R50 instrument, Canon Inc., USA)
ALSPAC children at ‘11-year visit’	5322	11.74 ± 0.23 (10.58–13.58)	2714 (51.0%)	-0.02 ± 1.12	Non-cycloplegic autorefraction (Canon R50 instrument, Canon Inc., USA)
ALSPAC children at ‘12-year visit’	5310	12.80 ± 0.23 (11.75–14.25)	2722 (51.3%)	-0.16 ± 1.17	Non-cycloplegic autorefraction (Canon R50 instrument, Canon Inc., USA)
ALSPAC children at ‘15-year visit’	4037	15.43 ± 0.28 (14.25–17.08)	2131 (52.8%)	-0.38 ± 1.28	Non-cycloplegic autorefraction (Canon R50 instrument, Canon Inc., USA)
ALSPAC adults	1476	44.22 ± 4.28 (32.00–59.00)	1476 (100.0%)	-0.59 ± 1.79	Non-cycloplegic autorefraction (Canon R50 instrument, Canon Inc., USA)

Abbreviations: BHAS = Busselton Healthy Ageing Study; ALSPAC = Avon Longitudinal Study of Parents and Children.

Table S4. Test for association of MMD grade and PGS for refractive error in the BHAS sample.

Logistic regression analysis for outcome MMD grade C3-C4 vs. MMD grade C0-C2 in the worse-affected eye of 4548 participants from the BHAS. Models were fit without (Model 1) and with (Model 2) inclusion of refractive error as a covariate.

	Model 1			Model 2		
**Parameter**	**Estimate**	**SE**	***P***	**Estimate**	**SE**	***P***
Intercept	-9.82	28.10	0.73	-3.88	28.50	0.89
**PGS**	**0.18**	**0.26**	**0.49**	**0.00**	**0.26**	**0.99**
Sex (reference: male)	0.01	0.53	0.99	0.07	0.54	0.89
Age	0.17	0.98	0.86	-0.05	0.99	0.96
Age^2	0.00	0.01	0.87	0.00	0.01	0.94
PC1	390.00	151.00	0.01	428.00	159.00	0.01
PC2	182.00	216.00	0.40	226.00	260.00	0.38
PC3	-53.60	170.00	0.75	-58.80	188.00	0.75
PC4	-15.50	365.00	0.97	20.40	381.00	0.96
PC5	31.10	81.50	0.70	43.90	85.20	0.61
PC6	-17.00	47.80	0.72	-12.40	50.70	0.81
PC7	100.00	144.00	0.49	81.80	145.00	0.57
PC8	59.20	91.20	0.52	56.70	97.80	0.56
PC9	22.60	63.10	0.72	22.80	66.20	0.73
PC10	-11.00	30.90	0.72	-4.27	30.90	0.89
**SphEq***	**-**	**-**	**-**	**-0.33**	**0.10**	**0.0007**

*SphEq = Refractive error in eye with worse MMD grade, or the average refractive error of the two eyes if the MMD grade in fellow eyes was equal.

Figure S1. Classification of ancestry groups.

Genetic principal components 1 and 2 (PC1 and PC2) were used to classify UK Biobank participants as East Asian (EAS), South Asian (SAS), African (AFR) or a “relaxed” definition of European (EUR). Individuals not classified in any of these groups (Mixed) were not included in the current analyses.

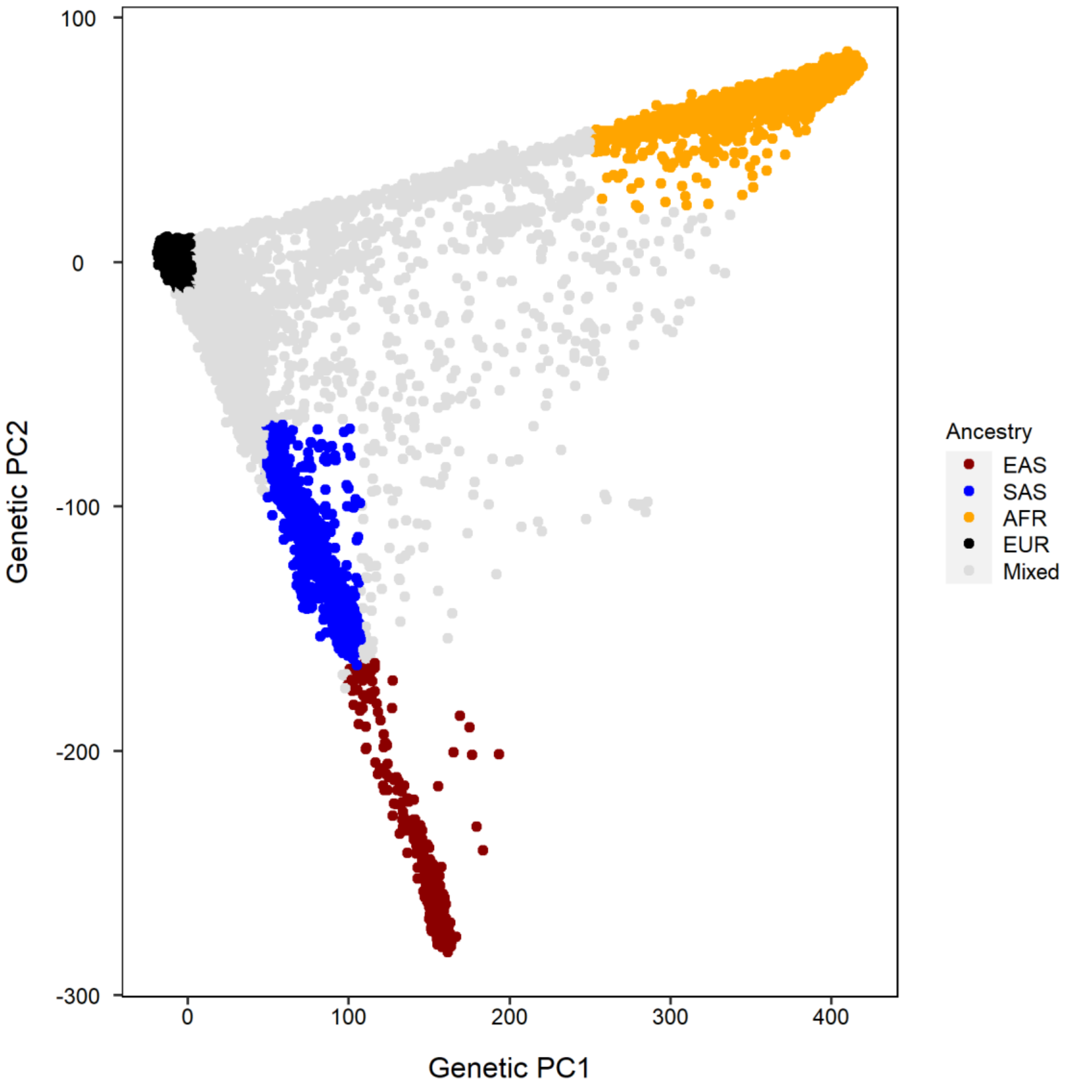

Figure S2. Selection Scheme for UK Biobank participants.

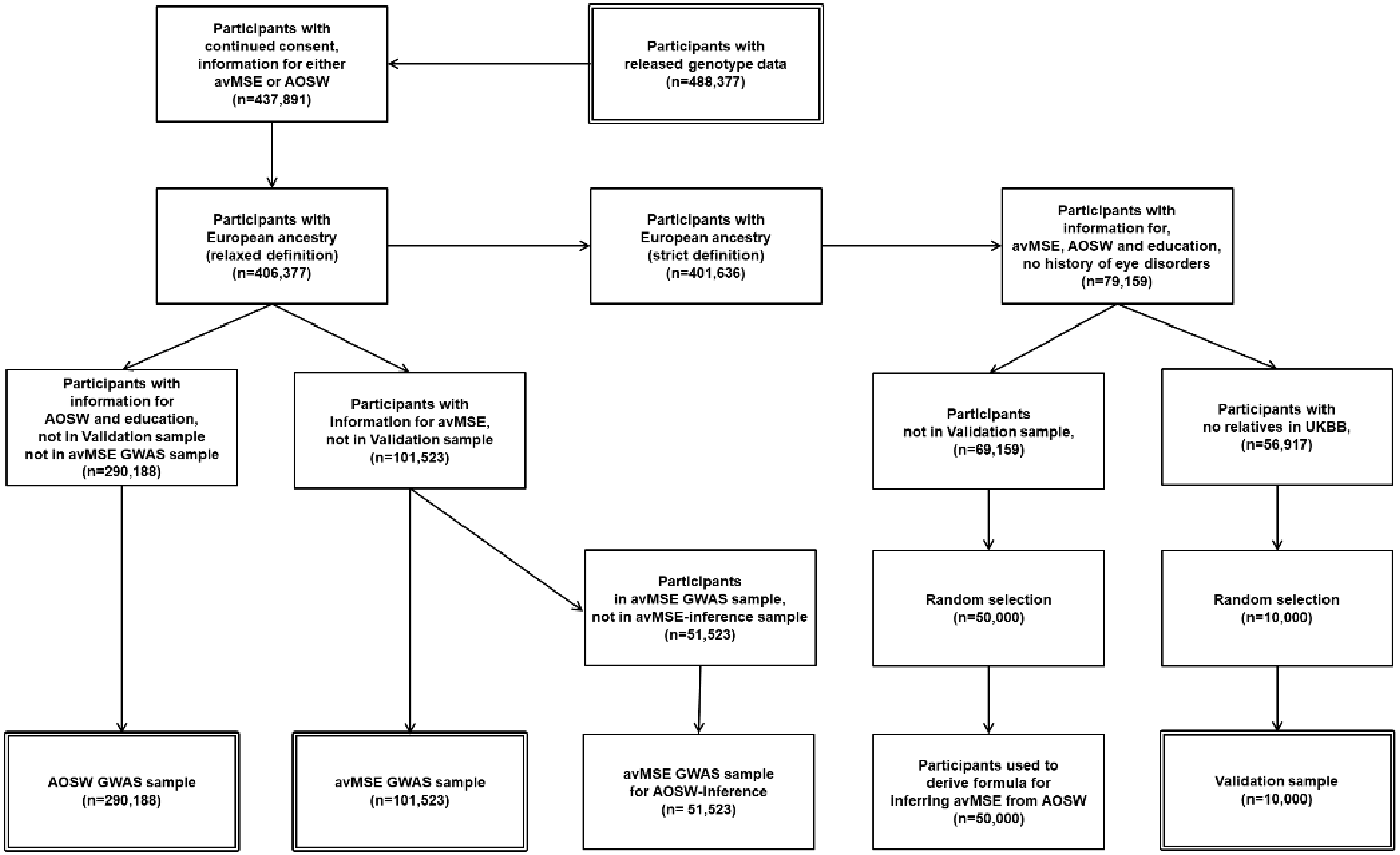

Figure S3. Flow diagram describing the leave-one-chromosome-out (LOCO) scheme.

The LOCO scheme was used to derive a set of PGSs "PGS_{loco}" to improve inference of *avSER* from *AOSW*. From a GWAS for *avSER* in 51 523 participants using the --predBetasFile function of BOLT, twenty-two separate PGSs were derived, each leaving one chromosome out (LOCO).

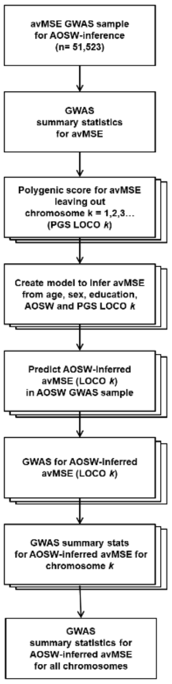

References

1. Tedja MS, Wojciechowski R, Hysi PG, et al. Genome-wide association meta-analysis highlights light-induced signaling as a driver for refractive error. *Nat Genet* 2018;50:834–48.
2. Magi R, Morris AP. GWAMA: software for genome-wide association meta-analysis. *BMC Bioinformatics* 2010;11:288.

CREAM Consortium members

Institution	Name
Bristol Medical School, Bristol, UK	Cathy Williams
Capital Medical University, Beijing, China	Shi-Ming Li
	Ningli Wang
	Ya Xing Wang
	Wen Bin Wei
Cardiff University, Cardiff, UK	Jeremy A. Guggenheim
Case Western Reserve University, Cleveland, Ohio, USA	Robert P. Igo
	Sudha K. Iyengar
	Jonathan H. Lass
Central Hospital of Central Finland, Jyväskylä, Finland	Olavi Pärssinen
DUKE-National University of Singapore, Singapore	Qiao Fan
Erasmus Medical Center, Rotterdam, The Netherlands	Annechien E.G. Haarman
	Adriana I. Iglesias
	Milly S. Tedja
	J. Willem L. Tideman
	Cornelia M. van Duijn
	Virginie J.M. Verhoeven
Flinders University, Adelaide, Australia	Jamie E. Craig
Helmholtz Zentrum München, Neuherberg, Germany	Konrad Oexle
Johannes Gutenberg University Mainz, Mainz, Germany	Stefan Nickels
	Norbert Pfeiffer
Johns Hopkins Medical Institutions, Baltimore, Maryland, USA	Robert Wojciechowski
King's College London, London, UK	Christopher J. Hammond
	Pirro G. Hysi
	Katie M. Williams
Kyoto University Graduate School of Medicine,, Kyoto, Japan	Masahiro Miyake
	Akitaka Tsujikawa
National Eye Institute/National Institutes of Health, Bethesda, USA	Emily Y. Chew
National Human Genome Research Institute, Bethesda, Maryland, USA	Joan E. Bailey-Wilson
	Deyana Lewis
	Qing Li
	Candace Middlebrooks
	Anthony Musolf
	Claire L. Simpson
National Research Council of Italy, Pavia, Italy	Ginevra Biino
National University of Singapore, Singapore	Ching-Yu Cheng
	E-Shyong Tai
Otsu Red Cross Hospital, Nagara, Japan	Kenji Yamashiro
QIMR Berghofer Medical Research Institute, Brisbane, Australia	Puya Gharahkhani
	Xikun Han
	Stuart MacGregor

	Nicholas G. Martin
Ruprecht-Karls-University of Heidelberg, Mannheim, Germany	Jost B. Jonas
Singapore Eye Research Institute, Singapore	Veluchamy A. Barathi
	Quan Hoang
	Seang-Mei Saw
Sun Yat-sen University, Guangzhou, China	Xiaohu Ding
	Xiaobo Guo
The Chinese University of Hong Kong, Hong Kong	Li Jia Chen
	Chi Pui Pang
	Jason C.S. Yam
The Hong Kong Polytechnic University, Hong Kong	Maurice K.H. Yap
	Shea Ping Yip
UCL Institute of Ophthalmology, London, UK	Paul J. Foster
	Anthony P. Khawaja
Université de Bordeaux, Bordeaux, France	Cécile Delcourt
University College London, London, UK	Jugnoo S. Rahi
University Hospital 'San Giovanni di Dio', Cagliari, Italy	Maurizio Fossarello
University of Edinburgh, Edinburgh, UK	Harry Campbell
	Caroline Hayward
	Igor Rudan
	Veronique Vitart
	James F. Wilson
University of Helsinki, Helsinki, Finland	Jaakko Kaprio
	Juho Wedenoja
University of Melbourne, Melbourne, Australia	Paul N. Baird
	Mingguang He
	Alex W. Hewitt
	David A. Mackey
	Srujana Sahebjada
University of Pennsylvania, Philadelphia, Pennsylvania, USA	Dwight Stambolian
University of Split, Soltanska 2, Split, Croatia	Ozren Polasek
University of Tampere, Tampere, Finland	Mika Kähönen
	Terho Lehtimäki
	Leo-Pekka Lyytikäinen
University of Tartu, Tartu, Estonia	Toomas Haller
	Andres Metspalu
University of Tasmania, Hobart, Australia	Kathryn P. Burdon
University of Toronto, Toronto, Ontario, Canada	Andrew D. Paterson
University of Turku, Turku, Finland	Olli Raitakari
University of Utah, Salt Lake City, Utah, USA	Margaret M. Deangelis
University of Western Australia, Perth, Australia	Seyhan Yazar
University of Wisconsin–Madison, Madison, Wisconsin, USA	Barbara E. Klein
	Kris Lee

	Terri L. Young
Wenzhou Medical University, Wenzhou, China	Xiangtian Zhou
Yokohama City University School of Medicine, Yokohama, Kanagawa, Japan	Nobuhisa Mizuki
	Akira Meguro

UK Biobank Eye and Vision Consortium members

Institution	Name
Cardiff University, Cardiff, UK	Jeremy Guggenheim
	James Morgan
Gloucestershire Hospitals NHS Foundation Trust, Gloucester, UK	Irene Stratton
King's College London, London, UK	Catey Bunce
	Eoin O'Sullivan
	Chris Hammond
	Pirro Hysi
	Katie Williams
Kingston University, London, UK	Sarah Barman
Leeds Teaching Hospitals NHS Trust, Leeds, UK	Martin McKibbin
Moorfields Eye Hospital, London, UK	Michelle Chan
	Alexander Day
	Parul Desai
	Cathy Egan
	Dan Gore
	Anthony Khawaja
	Gerassimos Lascaratos
	Praveen Patel
	Sobha Sivaprasad
	Nicholas Strouthidis
	Dhanes Thomas
	Adnan Tufail
	Ananth Viswanathan
Newcastle University, Newcastle, UK	David Steel
Queen's University Belfast, Belfast, UK	Bernadette Mcguinness
	Euan Paterson
	Gareth McKay
	Tunde Peto
	Jayne Woodside
	Usha Chakravarthy
	Ruth Hogg
St George's, University of London, London, UK	Chris Owen
	Alicja Rudnicka
	Robyn Tapp
UCL Institute of Child Health, London, UK	Jugnoo Rahi
UCL Institute of Neurology, London, UK	Axel Petzold
UCL Institute of Ophthalmology, London, UK	Sharon Chua
	Valentina Cipriani
	Paul Foster
	Marcus Fruttiger
	David Garway-Heath

	Alison Hardcastle
	Pearse A Keane
	Peng Tee Khaw
	Phil Luthert
	Tony Moore
	Zaynah Muthy
	Nikolas Pontikos
	Caroline Thaung
University Hospital, Nottingham, Nottingham, UK	Stephen Vernon
University of Bristol, Bristol, UK	Denize Atan
	Andrew Dick
	Cathy Williams
University of Cambridge, Cambridge, UK	Jennifer Yip
University of Dundee, Dundee, UK	Alexander Doney
	Emanuele Trucco
University of East Anglia, Norwich, UK	Max Yates
University of Edinburgh, Edinburgh, UK	Bal Dhillon
	Tom MacGillivray
	Danny Mitry
	Cathie Sudlow
	Veronique Vitart
University of Leeds, Leeds, UK	Jenny Barrett
	Sarah Mackie
University of Liverpool, Liverpool, UK	Simon Harding
	Yalin Zheng
University of Manchester, Manchester, UK	Tariq Aslam
	Paul Bishop
	Graeme Black
	Panagiotis Sergouniotis
University of Oxford, Oxford, UK	Naomi Allen
	John Gallacher
	Thomas Littlejohns
University of Southampton, Southampton, UK	Roxana Carare
	Sarah Ennis
	Jane Gibson
	Andrew Lotery
	Jay Self
