# Supplementary material for: A new polygenic score for refractive error improves detection of children at risk of high myopia but not the prediction of those at risk of myopic macular degeneration
Source: eBioMedicine. 2023 Apr 11;91:104551. doi: 10.1016/j.ebiom.2023.104551 (PMC10203044; doi:10.1016/j.ebiom.2023.104551)
Supplement: CREAM Consortium list of names [file mmc2.docx]

**The CREAM Consortium**

| **First names** | **Surname** |
| --- | --- |
| Joan E. | Bailey-Wilson |
| Paul N. | Baird |
| Veluchamy A. | Barathi |
| Ginevra | Biino |
| Kathryn P. | Burdon |
| Harry | Campbell |
| Li Jia | Chen |
| Ching-Yu | Cheng |
| Emily Y. | Chew |
| Jamie E. | Craig |
| Margaret M. | Deangelis |
| Cécile | Delcourt |
| Xiaohu | Ding |
| Qiao | Fan |
| Maurizio | Fossarello |
| Paul J. | Foster |
| Puya | Gharahkhani |
| Jeremy A. | Guggenheim |
| Xiaobo | Guo |
| Annechien E.G. | Haarman |
| Toomas | Haller |
| Christopher J. | Hammond |
| Xikun | Han |
| Caroline | Hayward |
| Mingguang | He |
| Alex W. | Hewitt |
| Quan | Hoang |
| Pirro G. | Hysi |
| Adriana I. | Iglesias |
| Robert P. | Igo |
| Sudha K. | Iyengar |
| Jost B. | Jonas |
| Mika | Kähönen |
| Jaakko | Kaprio |
| Anthony P. | Khawaja |
| Barbara E. | Klein |
| Jonathan H. | Lass |
| Kris | Lee |
| Terho | Lehtimäki |
| Deyana | Lewis |
| Qing | Li |
| Shi-Ming | Li |
| Leo-Pekka | Lyytikäinen |
| Stuart | MacGregor |
| David A. | Mackey |
| Nicholas G. | Martin |
| Akira | Meguro |
| Andres | Metspalu |
| Candace | Middlebrooks |
| Masahiro | Miyake |
| Nobuhisa | Mizuki |
| Anthony | Musolf |
| Stefan | Nickels |
| Konrad | Oexle |
| Chi Pui | Pang |
| Olavi | Pärssinen |
| Andrew D. | Paterson |
| Norbert | Pfeiffer |
| Ozren | Polasek |
| Jugnoo S. | Rahi |
| Olli | Raitakari |
| Igor | Rudan |
| Srujana | Sahebjada |
| Seang-Mei | Saw |
| Claire L. | Simpson |
| Dwight | Stambolian |
| E-Shyong | Tai |
| Milly S. | Tedja |
| J. Willem L. | Tideman |
| Akitaka | Tsujikawa |
| Cornelia M. | van Duijn |
| Virginie J.M. | Verhoeven |
| Veronique | Vitart |
| Ningli | Wang |
| Ya Xing | Wang |
| Juho | Wedenoja |
| Wen Bin | Wei |
| Cathy | Williams |
| Katie M. | Williams |
| James F. | Wilson |
| Robert | Wojciechowski |
| Jason C.S. | Yam |
| Kenji | Yamashiro |
| Maurice K.H. | Yap |
| Seyhan | Yazar |
| Shea Ping | Yip |
| Terri L. | Young |
| Xiangtian | Zhou |
