# Supplementary material for: A new polygenic score for refractive error improves detection of children at risk of high myopia but not the prediction of those at risk of myopic macular degeneration
Source: eBioMedicine. 2023 Apr 11;91:104551. doi: 10.1016/j.ebiom.2023.104551 (PMC10203044; doi:10.1016/j.ebiom.2023.104551)
Supplement: UK Biobank Eye and Vision Consortium list of names [file mmc3.docx]

**The UK Biobank Eye and Vision Consortium**

| **First names** | **Surname** |
| --- | --- |
| Naomi | Allen |
| Tariq | Aslam |
| Denize | Atan |
| Sarah | Barman |
| Jenny | Barrett |
| Paul | Bishop |
| Graeme | Black |
| Catey | Bunce |
| Roxana | Carare |
| Usha | Chakravarthy |
| Michelle | Chan |
| Sharon | Chua |
| Valentina | Cipriani |
| Alexander | Day |
| Parul | Desai |
| Bal | Dhillon |
| Andrew | Dick |
| Alexander | Doney |
| Cathy | Egan |
| Sarah | Ennis |
| Paul | Foster |
| Marcus | Fruttiger |
| John | Gallacher |
| David | Garway-Heath |
| Jane | Gibson |
| Dan | Gore |
| Jeremy | Guggenheim |
| Chris | Hammond |
| Alison | Hardcastle |
| Simon | Harding |
| Ruth | Hogg |
| Pirro | Hysi |
| Pearse A | Keane |
| Peng Tee | Khaw |
| Anthony | Khawaja |
| Gerassimos | Lascaratos |
| Thomas | Littlejohns |
| Andrew | Lotery |
| Phil | Luthert |
| Tom | MacGillivray |
| Sarah | Mackie |
| Bernadette | McGuinness |
| Gareth | McKay |
| Martin | McKibbin |
| Danny | Mitry |
| Tony | Moore |
| James | Morgan |
| Zaynah | Muthy |
| Eoin | O'Sullivan |
| Chris | Owen |
| Praveen | Patel |
| Euan | Paterson |
| Tunde | Peto |
| Axel | Petzold |
| Nikolas | Pontikos |
| Jugnoo | Rahi |
| Alicja | Rudnicka |
| Jay | Self |
| Panagiotis | Sergouniotis |
| Sobha | Sivaprasad |
| David | Steel |
| Irene | Stratton |
| Nicholas | Strouthidis |
| Cathie | Sudlow |
| Robyn | Tapp |
| Caroline | Thaung |
| Dhanes | Thomas |
| Emanuele | Trucco |
| Adnan | Tufail |
| Stephen | Vernon |
| Ananth | Viswanathan |
| Veronique | Vitart |
| Cathy | Williams |
| Katie | Williams |
| Jayne | Woodside |
| Max | Yates |
| Jennifer | Yip |
| Yalin | Zheng |
